# pH-Universal Water Splitting Catalyst: Ru-Ni Nanosheet Assemblies

**DOI:** 10.1016/j.isci.2019.01.004

**Published:** 2019-01-05

**Authors:** Jian Yang, Qi Shao, Bolong Huang, Mingzi Sun, Xiaoqing Huang

**Affiliations:** 1 College of Chemistry, Chemical Engineering and Materials Science, Soochow University, Suzhou, Jiangsu 215123, China; 2 Department of Applied Biology and Chemical Technology, The Hong Kong Polytechnic University, Hung Hom, Kowloon, Hong Kong SAR

**Keywords:** Catalysis, Energy Materials, Materials Science

## Abstract

Although electrochemical water splitting is an effective and green approach to produce oxygen and hydrogen, the realization of efficient bifunctional catalysts that are stable in variable electrolytes is still a significant challenge. Herein, we report a three-dimensional hierarchical assembly structure composed of an ultrathin Ru shell and a Ru-Ni alloy core as a catalyst functioning under universal pH conditions. Compared with the typical Ir/C-Pt/C system, superior catalytic performances and excellent durability of the overall water splitting under universal pH have been demonstrated. The introduction of Ni downshifts the d-band center of the Ru-Ni electrocatalysts, modulating the surface electronic environment. Density functional theory results reveal that the mutually restrictive d-band interaction lowers the binding of (Ru, Ni) and (H, O) for easier O-O and H-H formation. The structure-induced eg-dz^2^ misalignment leads to minimization of surface Coulomb repulsion to achieve a barrier-free water-splitting process.

## Highlights

• A facile method for the synthesis of 3D hierarchical assembly Ru-Ni NA catalyst
• Superior catalytic reactivity and stability of water splitting in universal pH
• The introduction of Ni can modify the d-band and surface electronic environment
• Minimization of the surface Coulomb repulsion leads to barrier-free catalysis process

## Graphical Abstract

**Figure:**
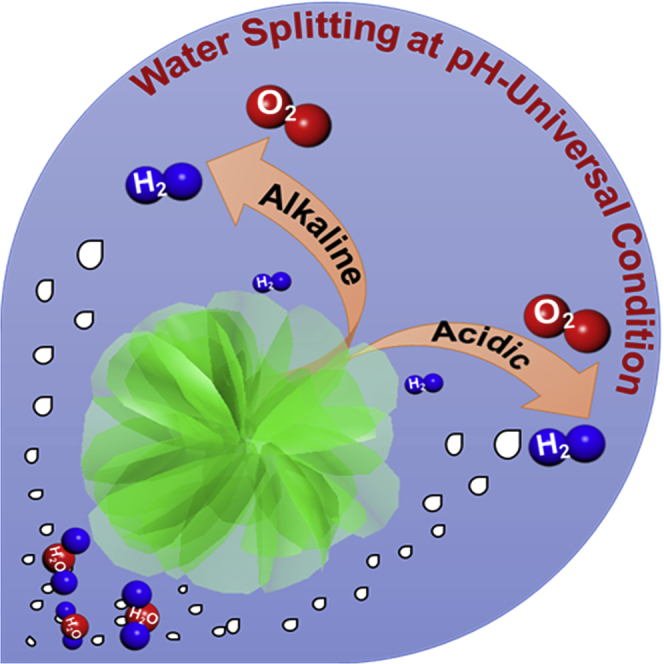

## Introduction

Hydrogen (H_2_) is becoming increasingly important as a future fuel compared with fossil fuels because of its advantages of clean and renewable energy generation (Dresselhaus and Thomas, 2001, Turner, 2004). Electrochemical water splitting provides an effective approach for H_2_ production. Water splitting consists of the hydrogen evolution reaction (HER) and oxygen evolution reaction (OER), both of which require efficient catalysts to reduce the overpotential for practical applications (Jin et al., 2015, Zhang et al., 2017a, Balogun et al., 2016). Although platinum (Pt) is regarded as a conventional HER catalyst in acidic solutions owing to its highest exchange current density and low Tafel slope, it shows an “incomparable” HER activity in alkaline solutions owing to the sluggish reaction kinetics (Ma et al., 2017, Mahmood et al., 2017, Zheng et al., 2016). Even though non-noble metal materials have been widely explored as enhanced catalysts for HER, the greatest challenge for the use of non-noble metal materials so far is that their HER activities still underperform Pt-based catalysts, and they are susceptible to acid corrosion (Zhang et al., 2017b, Conway and Tilak, 2002). Similar obstacles are still unavoidable for non-noble metal materials for OER applications owing to their relatively high overpotentials for driving the OER process and the low energy conversion efficiencies. To date, pursuit of effective catalysts for both OER and HER in the same electrolyte, not to mention under universal pH conditions, has been extremely desirable (Zheng et al., 2014, Wang et al., 2018a, Wang et al., 2018b, Ellis et al., 2010). Therefore, the development of efficient and stable bifunctional catalysts for the simultaneous production of H_2_ and oxygen (O_2_) under universal pH conditions is still a significant challenge.

It has been generally considered that noble metal materials, such as Ru-based catalysts, are the most promising catalysts for use as overall water-splitting catalysts owing to their promising activities for the two half-reactions in both acidic and alkaline solutions as well as their high stability under extreme conditions (Lu et al., 2014, Jin et al., 2016, Petrykin et al., 2010, Seitz et al., 2016, Kong et al., 2016). However, the water-splitting performances of the reported Ru-based catalysts are still far from satisfactory, particularly under universal pH conditions. From the viewpoint of the structure, a two-dimensional (2D) structure can provide great opportunities for enhancing the electrochemical performance because it largely exposes the surface area (Hang et al., 2014, Gao et al., 2012). However, undesirable drawbacks arise from the severe aggregation or fracture that usually occurs during the electrochemical process, inevitably leading to the obvious activity decay. This renders the conventional 2D structure not an ideal candidate for efficient electrocatalysis (Zheng et al., 2014, Chhowalla et al., 2013, Hwang et al., 2011, Chen et al., 2015). Based on this, the assembly of 2D structures into unique 3D structures may provide an effective strategy to achieve efficient catalysts for water splitting under universal pH conditions because the structures can achieve a large exposure of the active sites while stabilizing the structure.

## Results

### Synthesis and Characterization of Ru-Ni NAs

To surmount this challenge, we report an efficient wet chemical approach for the synthesis of 3D hierarchical Ru-Ni nanosheet assemblies (NAs) consisting of ultrathin nanosheets as subunits and explore their high performances for overall water splitting under universal pH conditions. The distinctive hierarchical NA structures are highly beneficial for enhancing electrochemical energy conversion. We found that the introduction of Ni into Ru largely downshifts the d-band center of the Ru-Ni NAs and effectively modulates the surface environment for HER. After air treatment at 350°C, the newly generated abundant RuO_2_ provides effective active sites for OER. As a result, the Ru-Ni NAs deliver high HER and OER activities as well as outstanding stability under a broad range of pH conditions. More interestingly, Ru_3_Ni_3_ NAs demonstrated potential applications for overall water splitting with a lower overpotential, smaller Tafel slope, and better stability than the reference Ir/C-Pt/C catalyst.

A typical preparation of Ru-Ni NAs was introduced by adding ruthenium(III) acetylacetonate (Ru(acac)_3_), nickel(II) acetylacetonate (Ni(acac)_2_), phloroglucinol, tetramethylammonium bromide, polyvinylpyrrolidone (PVP), and benzyl alcohol into a glass vial. After capping the vial, the mixture was ultrasonicated for approximately 1 h. The resulting homogeneous mixture was then heated from room temperature to 160°C and maintained at 160°C for 5 h using an oil bath. Ru-Ni NAs with different Ru/Ni ratios (i.e., Ru_3_Ni_3_ NAs, Ru_3_Ni_2_ NAs, and Ru_3_Ni_1_ NAs) have been readily achieved by tuning the Ru/Ni precursor amount ratios (Figures S1A–S1C).

**Figure 1:**
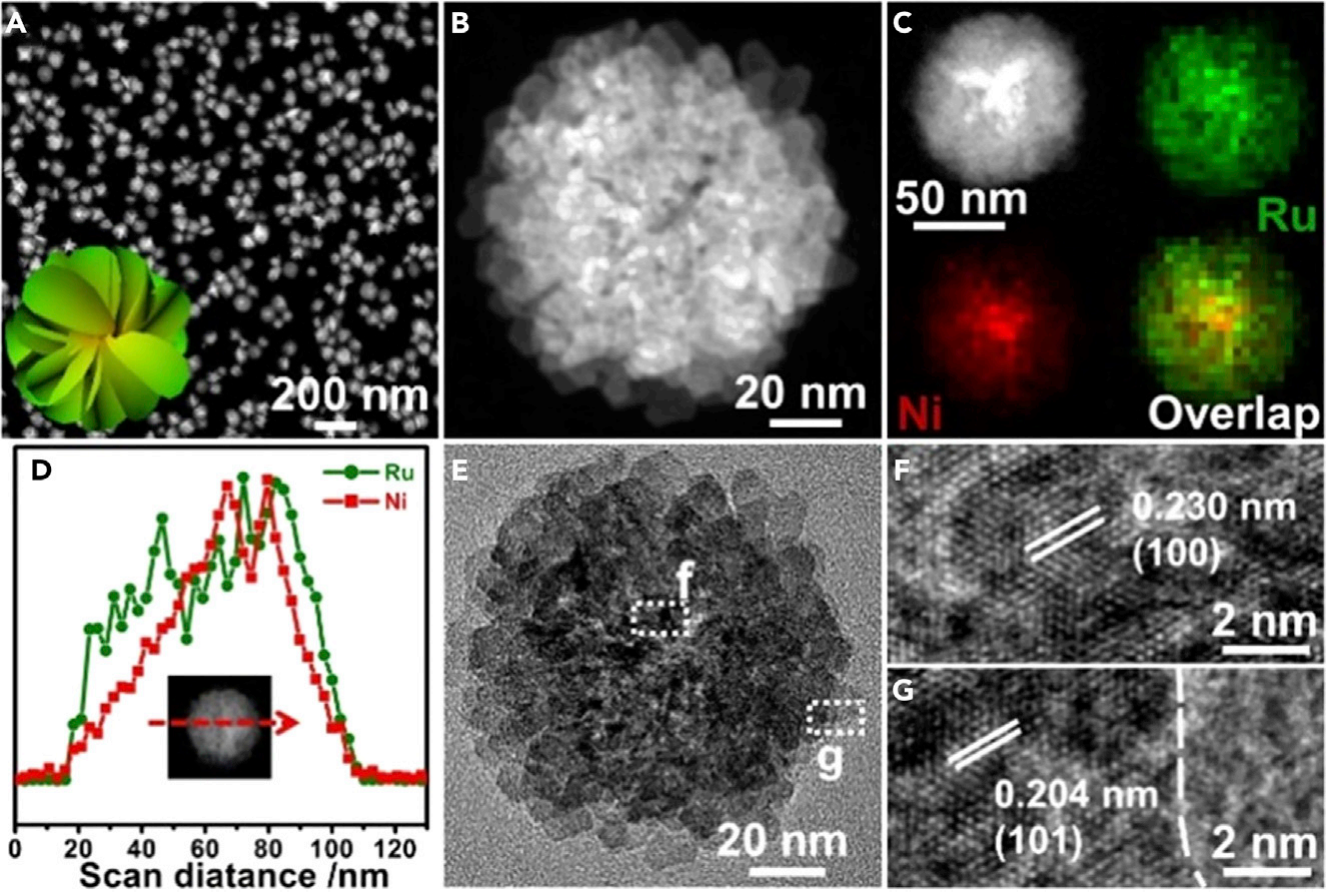
Structural Characterizations of the Ru_3_Ni_3_ NAs (A–G) (A and B) HAADF-STEM image, (C) HAADF-STEM image and elemental mapping, (D) line scans, (E) TEM image, and (F and G) high-resolution TEM images of the Ru_3_Ni_3_ NAs.

The detailed characterizations of Ru_3_Ni_3_ NAs were further carried out to determine the 3D assembly structure (Figures 1, S1D, and S1E). The high-angle annular dark-field scanning transmission electron microscopic (HAADF-STEM) image (Figure 1A) showed at first glance that all the products had a spherical outline, which indicated the high purity of product. For a close view of the Ru_3_Ni_3_ NAs, enlarged HAADF-STEM was performed, and a 3D flower-like structure assembled by hierarchical 2D nanosheet subunits was clearly observed (Figure 1B). Elemental mappings and line scans showed that the flower-like Ru_3_Ni_3_ NAs had a typical core-shell structure consisting of a Ru-Ni core and Ru shell (Figures 1C and 1D). Compared with those of the pure Ru NAs, additional X-ray diffraction (XRD) peaks in the Ru-Ni alloy were observed for 3D Ru_3_Ni_3_ NAs, which further confirmed the core-shell structure of the Ru_3_Ni_3_ NAs with the Ru phase and Ru-Ni alloy phase (Figures S1D and S1E). As revealed by the high-resolution transmission electron microscopic (TEM) image of the Ru_3_Ni_3_ NAs, lattice fringes with interplanar distances of 0.204 and 0.230 nm were observed, which correlated well with the (101) plane of Ru and the (100) plane of the Ru-Ni alloy, respectively (Figures 1E–1G).

Notably, the morphologies of Ru_3_Ni_2_ NAs and Ru_3_Ni_1_ NAs with different Ru/Ni ratios were similar (Figures S2A, S2B, S2E, S2H, S2I, and S2L). The XRD results show that as the amount of Ni increased, the main peaks of the Ru-Ni alloy approach the standard pure Ni XRD peaks (PCPDS No. 89–7,129), which suggested the successful alloying of Ni into Ru. The energy dispersive spectroscopy (EDS) elemental mapping images and line scans confirm that the alloys have a core-shell structure similar to that of the Ru_3_Ni_3_ NAs (Figures S2C, S2D, S2J, and S2K). The same lattice fringes with an interplanar distance of 0.204 nm were found in the Ru_3_Ni_2_ NAs and Ru_3_Ni_1_ NAs, which correlated well to the (101) plane of Ru. Lattice fringes of the (100) Ru-Ni alloy with interplanar distances of 0.231 and 0.232 nm were also observed in the Ru_3_Ni_2_ NAs and Ru_3_Ni_1_ NAs, respectively (Figures S2F, S2G, S2M, and S2N).

The direct creation of unique, 3D Ru-Ni superstructures with ultrathin building blocks is the most striking feature of the synthesis reported here, which has never been reported previously. To gain a better understanding of the growth mechanism behind the successful synthesis, characterizations of the intermediates collected at different reaction times were also carefully performed (Figures S3A–S3J). At the beginning of the reaction (25 min), intermediates with messy and irregular multi-branched structures were observed (Figures S3A and S3B). Nanosheets began to form, and a portion of the assembled flower-shaped intermediates appeared at a reaction time of 40 min (Figures S3C and S3D). When the reaction reached 1.5 h, the diameter of the flower-shaped intermediates increased (Figures S3E and S3F). After the reaction progressed for 3 h, the monodispersed, hierarchical assembly became obvious (Figures S3G and S3H). A further increase in the size of the Ru_3_Ni_3_ NAs was observed after the completion of the reaction (Figures S3I and S3J). The different reaction intermediates were also further analyzed by XRD (Figure S4), and the peaks of Ru and small peaks of the Ru-Ni alloy were detected during the initial 25 min. With the prolonged reaction time, the peak indexed to the Ru-Ni alloy became increasingly obvious and shifted to a higher angle, which indicated that more Ni was reduced and alloyed with Ru (Figure S3K).

To further understand the formation progress behind the successful synthesis, the effect of various experimental parameters on the morphology of Ru-Ni NAs was carried out. The results reveal that the combined use of PVP, phloroglucinol, and tetramethylammonium bromide was essential for the successful creation of Ru-Ni NAs. The Ru-Ni NAs could not be obtained in the absence of any PVP or phloroglucinol (Figures S5A, S5B, S7A, and S7B). Further detailed control experiments show that high-quality Ru-Ni NAs could only be obtained in the presence of specific amount of phloroglucinol and tetramethylammonium bromide. For example, irregular morphology was obtained when the amounts of phloroglucinol and tetramethylammonium bromide were changed (Figures S5 and S6), and a layered product with low yield was obtained when benzyl alcohol was replaced by ethylene glycol (Figures S7C and S7D). The morphology of assemblies has changed greatly without using Ni(acac)_2_ (Figure S8).

### HER Performance of Ru-Ni NAs

Considering that Ru is expected to have high activities for HER and OER, the design of Ru-based catalysts for overall water splitting is highly significant from the viewpoint of practical applications (Pu et al., 2017, Jiang et al., 2015), but the systematic study of Ru-based catalysts is still very limited, especially in a broad pH range. To this end, detailed HER and OER measurements were carried out in electrolytes with different pH values using Ru-Ni NAs as the candidate catalyst. All electrochemical measurements were performed in a standard three-electrode system with a saturated calomel electrode as the reference electrode and a carbon rod as the counter electrode. The reference electrodes were calibrated before the electrochemical measurements (Figure S9). All polarization curves were recorded without iR compensation. Before the electrocatalytic measurements, all different Ru-Ni NAs were loaded on a carbon support (Vulcan XC72R carbon) by sonication. Ru loading of 20 wt % was maintained in each catalyst, and no obvious morphological changes were observed after heat treatment (Figure S10). The resulting Ru-Ni NAs/C were then dispersed in a mixture solvent containing isopropanol and Nafion (5%) and sonicated for 30 min to form a homogeneous catalyst ink. The concentration of the Ru-Ni NAs loading on the carbon powder was controlled at 2 mg mL^−1^; 10 μL catalyst ink was uniformly dropped onto a glassy carbon electrode and dried naturally at room temperature.

**Figure 2:**
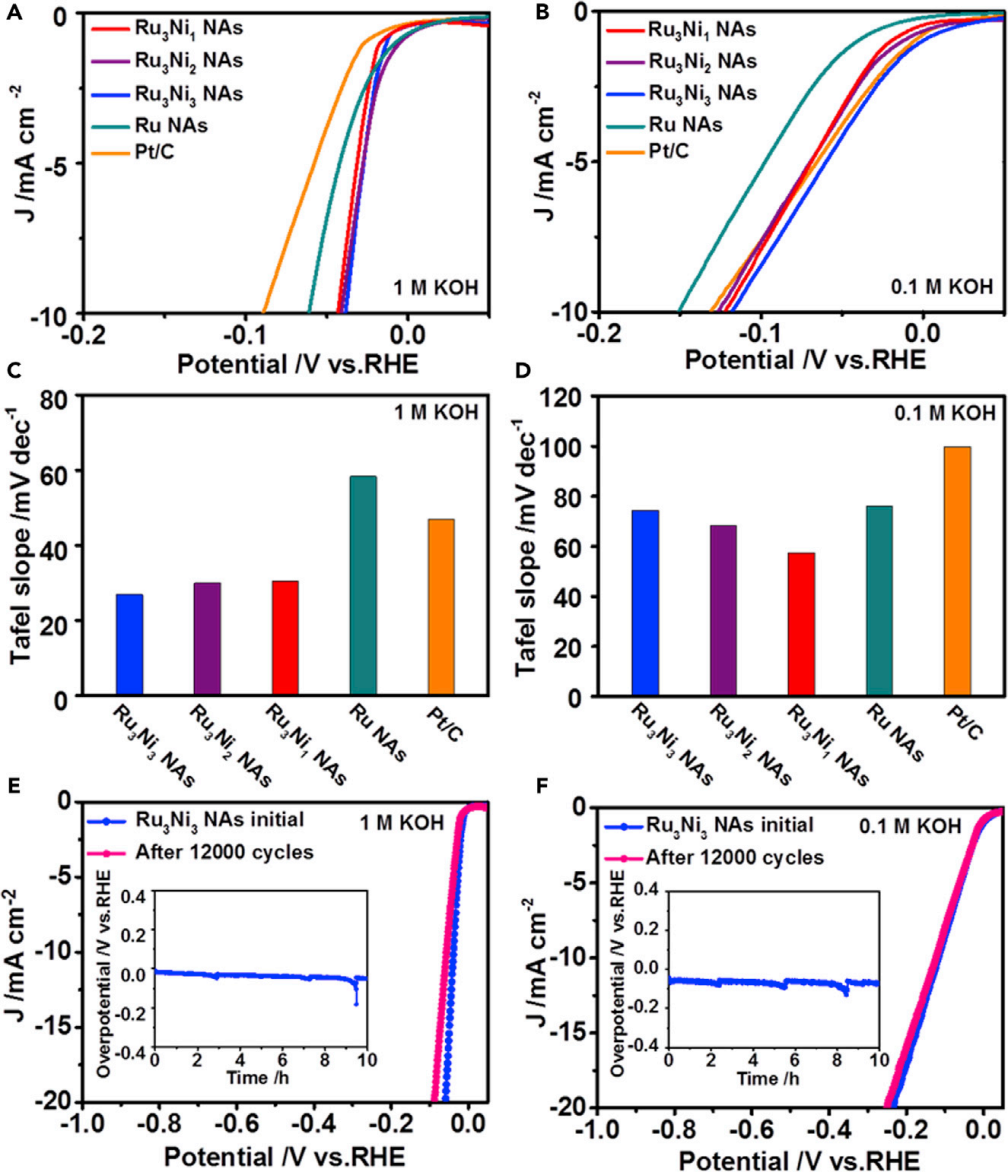
HER Performances of the Ru_3_Ni_3_ NAs, Ru_3_Ni_2_ NAs, Ru_3_Ni_1_ NAs, Ru NAs, and Pt/C under Different Alkaline Conditions (A and C) (A) The polarization curves and (C) corresponding Tafel plots in 1 M KOH. (B and D–F) (B) The polarization curves and (D) corresponding Tafel plots in 0.1 M KOH. Scan rate is 5 mV s^−1^. Polarization curves of the Ru_3_Ni_3_ NAs before and after 12,000 cycles in (E) 1 M KOH and (F) 0.1 M KOH solutions at a scan rate of 5 mV s^−1^. Insets: chronopotentiometry curves of the Ru_3_Ni_3_ NAs in 1 M KOH and 0.1 M KOH solutions at a constant current density of 5 mA cm^−2^.

The HER performance of the Ru-Ni NAs/C was first explored at a slow scan rate of 5 mV s^−1^ to ensure steady-state behavior on the electrode surface. To obtain the best performance of the Ru-Ni NAs/C in HER, we first determined the effects of the annealing temperature and atmosphere on HER performance by using Ru_3_Ni_3_ NAs as the candidate material. As shown in Figures S11A and S11B, the sample annealed at 250°C for 1 h exhibited the best HER activity in both alkaline and acidic conditions (0.5 M H_2_SO_4_ and 1 M KOH solutions). Figure 2A shows the polarization curves of the Ru-Ni NAs and Ru NAs and commercial Pt/C in 1 M KOH. In detail, at a current density of 10 mA cm^−2^, the overpotentials of Ru_3_Ni_3_ NAs, Ru_3_Ni_2_ NAs, Ru_3_Ni_1_ NAs, Ru NAs, and commercial Pt/C were 39, 42, 44, 62, and 90 mV, respectively, versus the reversible hydrogen electrode (RHE), and the Ru_3_Ni_3_ NAs showed the smallest value. The Tafel slope is an intrinsic property of the catalyst that is determined by the rate-limiting step of the HER (Cherevko et al., 2016). Importantly, the Tafel slopes of the Ru_3_Ni_3_ NAs, Ru_3_Ni_2_ NAs, and Ru_3_Ni_1_ NAs were calculated to be 26.9, 29.9, and 30.5 mV dec^−1^, respectively (Figures 2C and S12A). In contrast, the Ru NAs and commercial Pt/C showed relatively high Tafel slopes of 58.3 mV dec^−1^ and 46.8 mV dec^−1^. The electrocatalytic stability of the Ru_3_Ni_3_ NAs was further studied by both long-term cycling and chronopotentiometry tests, and the polarization curves of Ru_3_Ni_3_ NAs exhibited no obvious change after 12,000 cycles (Figure 2E). The Ru_3_Ni_3_ NAs showed only a slight potential increase after 10 h of chronopotentiometry at a current density of 5 mA cm^−2^ (Figure 2E, inset).

With the change in the electrolyte to 0.1 M KOH, the Ru-Ni NAs still showed promising HER activities. At 10 mA cm^−2^, the overpotentials of the Ru_3_Ni_3_ NAs, Ru_3_Ni_2_ NAs, Ru_3_Ni_1_ NAs, Ru NAs, and commercial Pt/C were 119, 127, 123, 152, and 132 mV, respectively (Figure 2B). In addition to the low overpotentials, the Ru_3_Ni_3_ NAs, Ru_3_Ni_2_ NAs, and Ru_3_Ni_1_ NAs also exhibited lower Tafel slopes than Pt/C (99.7 mV dec^−1^) and Ru NAs (76.0 mV dec^−1^) (Figures 2D and S12B). The Ru_3_Ni_3_ NAs also exhibited excellent durability after 12,000 cycles and in the chronopotentiometry test in 0.1 M KOH (Figure 2F), which indicated that the Ru_3_Ni_3_ NAs exhibit a superior HER activity and durability under alkaline conditions.

The HER properties of the Ru-Ni NAs under acidic conditions were further investigated. Figure S13 shows that the overpotentials of the Ru_3_Ni_3_ NAs, Ru_3_Ni_2_ NAs, Ru_3_Ni_1_ NAs, and Ru NAs were 39 and 96 mV, 39 and 115 mV, 46 and 112 mV, and 55 and 122 mV at a current density of 10 mA cm^−2^ in 0.5 M H_2_SO_4_ and 0.05 M H_2_SO_4_, respectively. The Tafel slopes of the Ru_3_Ni_3_ NAs, Ru_3_Ni_2_ NAs, Ru_3_Ni_1_ NAs, and Ru NAs were 53.9 and 67.1 mV dec^−1^, 53.5 and 64.0 mV dec^−1^, 54.2 and 66.8 mV dec^−1^, and 81.6 and 79.6 mV dec^−1^ in 0.5 M H_2_SO_4_ and 0.05 M H_2_SO_4_, respectively. The Ru-Ni NAs showed a much better HER performance than the Ru NAs, indicating the vital role of Ni in improving the HER performance. After the working electrode was cycled for 6,000 cycles, the Ru_3_Ni_3_ NAs exhibited the best durability under acidic conditions with potential increases of only 62 and 39 mV in 0.5 M H_2_SO_4_ and 0.05 M H_2_SO_4_, respectively. In addition, after the 12-h chronopotentiometry test at 5 mA cm^−2^ in 0.5 M H_2_SO_4_ and 0.05 M H_2_SO_4_, the Ru_3_Ni_3_ NAs showed only potential increases of 36 and 49 mV, respectively (Figures S13E and S13F).

### OER Performances of Ru-Ni NAs

The obtained Ru-Ni NAs were also successfully applied as efficient OER catalysts. Before the OER tests, the Ru-Ni NAs were also subjected to thermal annealing in air at different temperatures because Ru oxide has been discovered to be an active component for the OER (Petrykin et al., 2010, Reier et al., 2012). As shown in Figures S11C and S11D, the catalyst after heat treatment in air (350°C, 2 h) showed the best performance under both acidic and alkaline conditions (0.5 M H_2_SO_4_ and 1 M KOH). The TEM images show that the hierarchical structures were largely preserved (Figures S10C and S10D). We also studied the structural characterization of NAs after heat treatment by STEM image, elemental mapping, and line scan, where the core-shell structures of Ru_3_Ni_3_ NAs are largely reserved (Figure S14). We also showed that the carbon can enhance both the electrical conductivity and the dispersion of Ru_3_Ni_3_ NAs, and thus improve the electrocatalysis (Figure S15). To evaluate the OER performances of Ru-Ni under universal pH conditions, we tested the OER performances in both acidic (0.5 and 0.05 M H_2_SO_4_) and alkaline (1 and 0.1 M KOH) electrolytes. The commercial Ir/C catalyst was chosen as the reference because Ir is considered to be the benchmark catalyst for OER (Lettenmeier et al., 2016, Zhang et al., 2017c).

**Figure 3:**
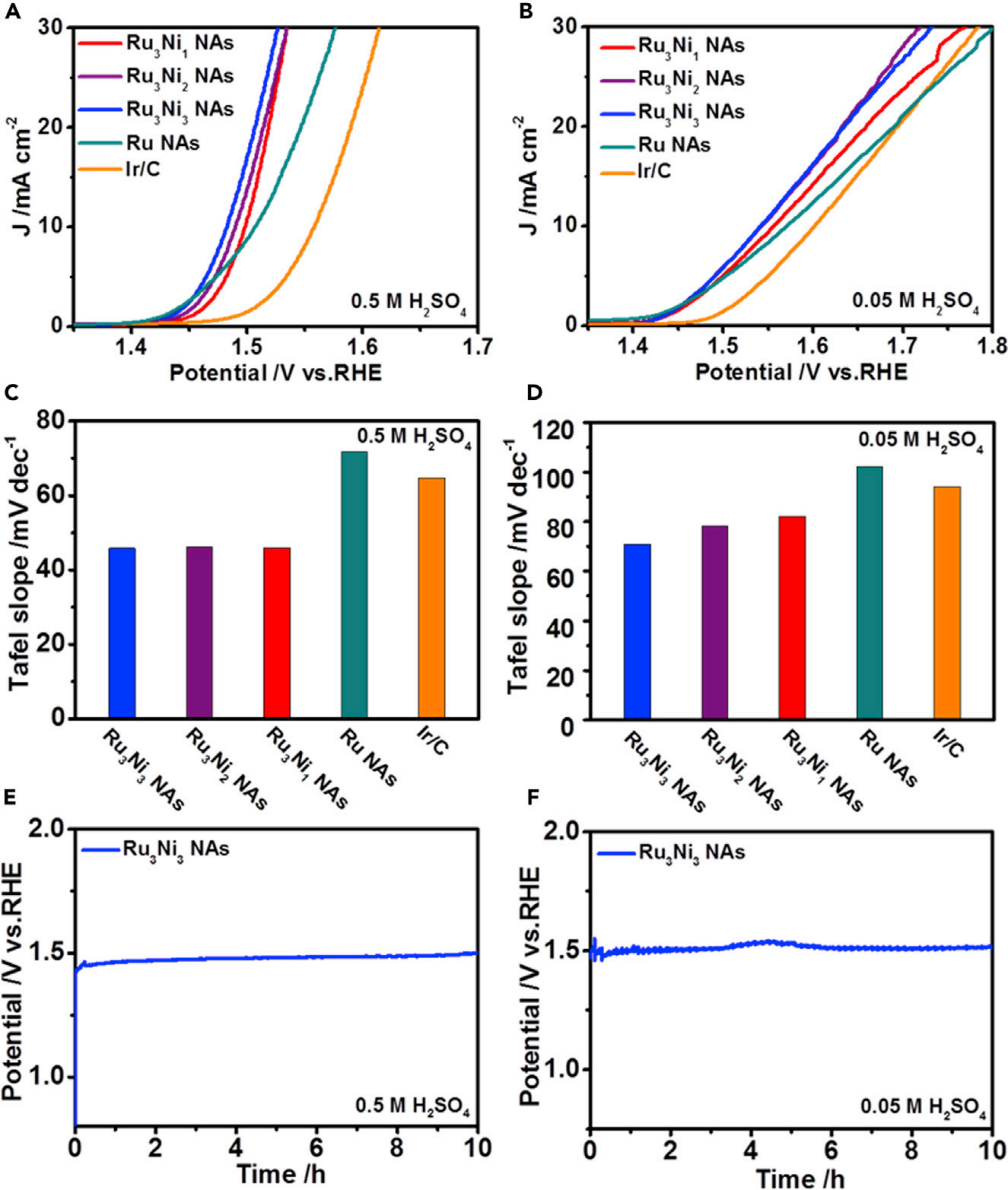
OER Performances of the Ru_3_Ni_3_ NAs, Ru_3_Ni_2_ NAs, Ru_3_Ni_1_ NAs, Ru NAs, and Commercial Ir/C under Different Acidic Conditions (A and C) (A) The polarization curves and (C) corresponding Tafel plots in 0.5 M H_2_SO_4_. (B and D–F) (B) The polarization curves and (D) corresponding Tafel plots in 0.05 M H_2_SO_4_. Scan rate is 5 mV s^−1^. Chronopotentiometry curves of the Ru_3_Ni_3_ NAs in (E) 0.5 M H_2_SO_4_ and (F) 0.05 M H_2_SO_4_ solutions at a current density of 5 mA cm^−2^.

Examination of the OER polarization curves in 0.5 and 0.05 M H_2_SO_4_ shows that the Ru-Ni NAs showed much better OER activities than the Ru NAs and commercial Ir/C. To drive a current density of 10 mA cm^−2^, the Ru_3_Ni_3_ NAs, Ru_3_Ni_2_ NAs, and Ru_3_Ni_1_ NAs required overpotentials of 252 mV, 260 mV, and 268 mV in 0.5 M H_2_SO_4_, respectively (Figure 3A). The Tafel slopes of the Ru_3_Ni_3_ NAs, Ru_3_Ni_2_ NAs, and Ru_3_Ni_1_ NAs derived from Figure 3A were 45.8, 46.1, and 46.0 mV dec^−1^ in 0.5 M H_2_SO_4_, respectively. In contrast, the commercial Ir/C and Ru NAs required larger overpotentials of 328 and 277 mV in 0.5 M H_2_SO_4_, respectively. The Tafel slopes of the commercial Ir/C and Ru NAs were also larger than those of the Ru-Ni NAs (Figures 3C and S16A). Similar trends were also obtained in 0.05 M H_2_SO_4_, and the Ru_3_Ni_3_ NAs showed the lowest overpotential and Tafel slope of 312 mV and 70.8 mV dec^−1^, respectively (Figures 3B, 3D, and 6B).

We further measured the OER activities in different alkaline electrolytes. The overpotentials of the Ru_3_Ni_3_ NAs, Ru_3_Ni_2_ NAs, and Ru_3_Ni_1_ NAs were 304, 309, and 301 mV in 1 M KOH, whereas the Ru NAs and commercial Ir/C showed larger overpotentials of 351 and 311 mV, respectively (Figure S17A). The Tafel slopes of the Ru_3_Ni_3_ NAs, Ru_3_Ni_2_ NAs, Ru_3_Ni_1_ NAs, Ru NAs, and commercial Ir/C derived from Figure S15A were 91.7, 67.9, 73.4, 111.1, and 47.1 mV dec^−1^, respectively (Figure S17C). When the solution is replaced by a dilute alkaline solution (0.1 M KOH), in which it is more difficult for the OER to proceed (Lu and Zhao, 2015), the Ru-Ni NAs also exhibited a high activity. The overpotentials of the Ru_3_Ni_3_ NAs, Ru_3_Ni_2_ NAs, and Ru_3_Ni_1_ NAs were 394, 390, and 384 mV, respectively, which were smaller than those of the Ru NAs (439 mV) and commercial Ir/C (407 mV) (Figure S17B). The Tafel slopes of the Ru_3_Ni_3_ NAs, Ru_3_Ni_2_ NAs, Ru_3_Ni_1_ NAs, Ru NAs, and commercial Ir/C derived from Figure S17B were 133.8, 131.4, 130.2, 140.7, and 111.1 mV dec^−1^, respectively (Figure S17D). All these results confirmed that the unique Ru-Ni NAs show excellent OER performances compared with the Ru NAs. In addition, in the 10-h chronopotentiometry test, the Ru_3_Ni_3_ NAs showed limited degradation after continuous electrolysis at 5 mA cm^−2^ in 0.5 M H_2_SO_4_, 0.05 M H_2_SO_4_, 1 M KOH, and 0.1 M KOH (Figures 3E, 3F,S17E, and S17F). No obvious morphological changes were observed in 0.5 M H_2_SO_4_ and 1 M KOH after the chronopotentiometry test (Figure S18), which demonstrated that the Ru-Ni NAs are indeed “acidic- and alkaline-stable” OER catalysts. To further demonstrate the OER and HER stability, chronopotentiometry test at higher current density was also performed, where the Ru_3_Ni_3_ NAs still showed limited degradations after continuous OER and HER electrolysis at 10 mA cm^−2^ in 0.5 M H_2_SO_4_ and 1 M KOH (Figure S19).

### Water-Splitting Performance of the Ru-Ni NAs

**Figure 4:**
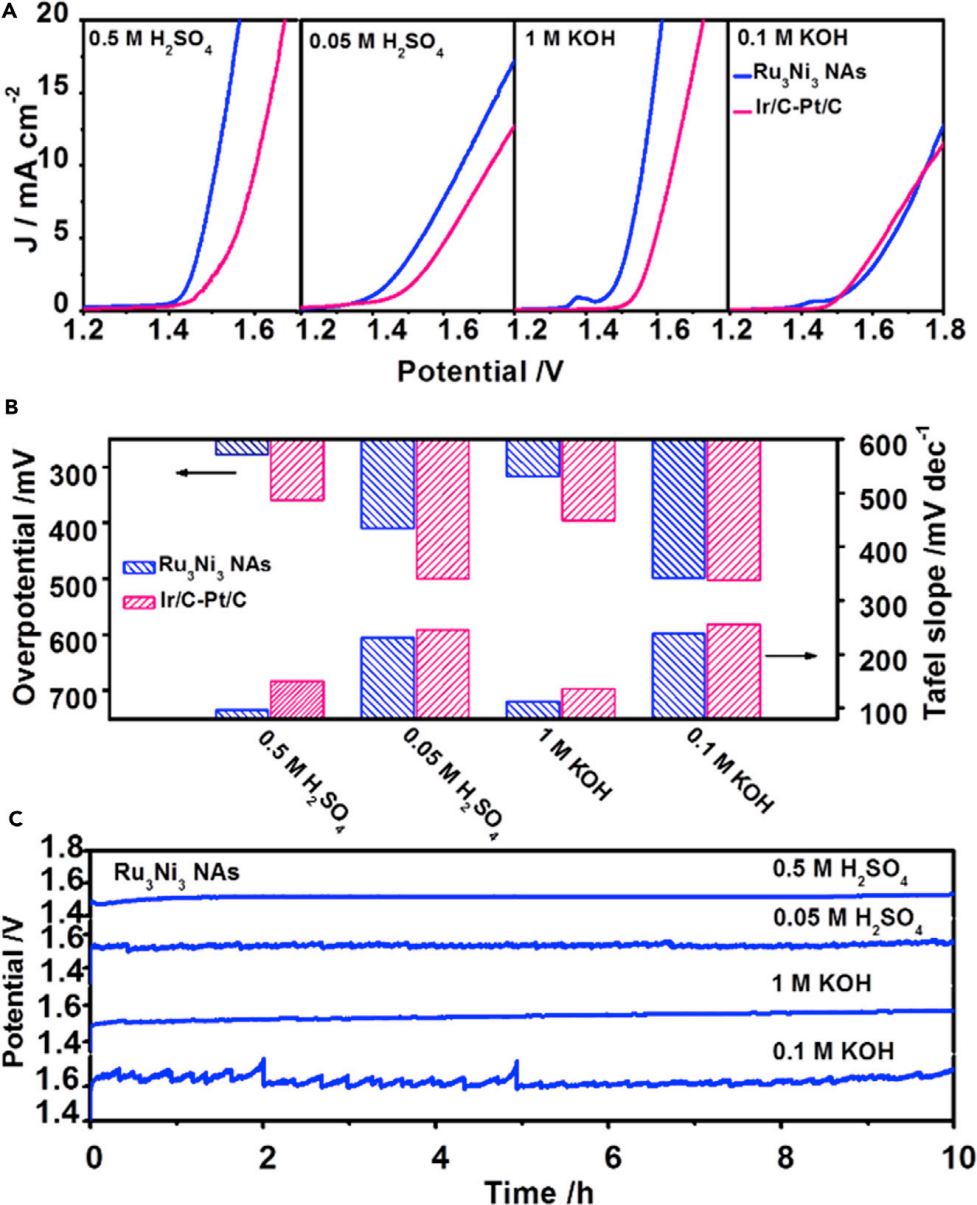
Overall Water Splitting Performances of the Ru-Ni NAs under Different pH Conditions (A and B) (A) Polarization curves for the overall water splitting and (B) the overpotentials and Tafel plots of the Ru_3_Ni_3_ NAs and Ir/C-Pt/C in 0.5 M H_2_SO_4_, 0.05 M H_2_SO_4_, 1 M KOH, and 0.1 M KOH. (C) Chronopotentiometry curves of the Ru_3_Ni_3_ NAs in 0.5 M H_2_SO_4_, 0.05 M H_2_SO_4_, 1 M KOH, and 0.1 M KOH at a constant current density of 5 mA cm^−2^.

As we explored the best catalysts for HER and OER under both acidic and alkaline conditions, a two-electrode setup with anodic catalyst Ru_3_Ni_3_ NAs after air treatment at 250°C for 1 h and cathodic catalyst Ru_3_Ni_3_ NAs after air treatment at 350°C for 2 h was used to study the potential application of Ru-Ni NAs in overall water splitting under universal pH conditions. The Linear Sweep Voltammetry (LSV) plots of Ru_3_Ni_3_ NAs and Ir/C-Pt/C under different pH conditions are presented in Figure 4A. The data clearly show that both the potentials and Tafel slopes of the Ru_3_Ni_3_ NAs are much lower than those of Ir/C-Pt/C. The Ru_3_Ni_3_ NAs show an overpotential of 280 mV in 0.5 M H_2_SO_4_, which is considerably lower than that of Ir/C-Pt/C (370 mV). The Tafel slope of the Ru_3_Ni_3_ NAs is only 96.9 mV dec^−1^, whereas that of Ir/C-Pt/C is as high as 150.1 mV dec^−1^ (Figures 4B and S20A), indicating that the reaction kinetics of the Ru_3_Ni_3_ NAs are much faster than those of Ir/C-Pt/C. Significantly, the Ru_3_Ni_3_ NAs showed excellent durability with limited degradation after a 10-h chronopotentiometry test at 5 mA cm^−2^ in 0.5 M H_2_SO_4_, 0.05 M H_2_SO_4_, 1 M KOH, and 0.1 M KOH (Figure 4C). Overall, these results confirmed that the Ru-Ni NAs can serve as excellent water-splitting catalysts under universal pH conditions.

## Discussions

**Figure 5:**
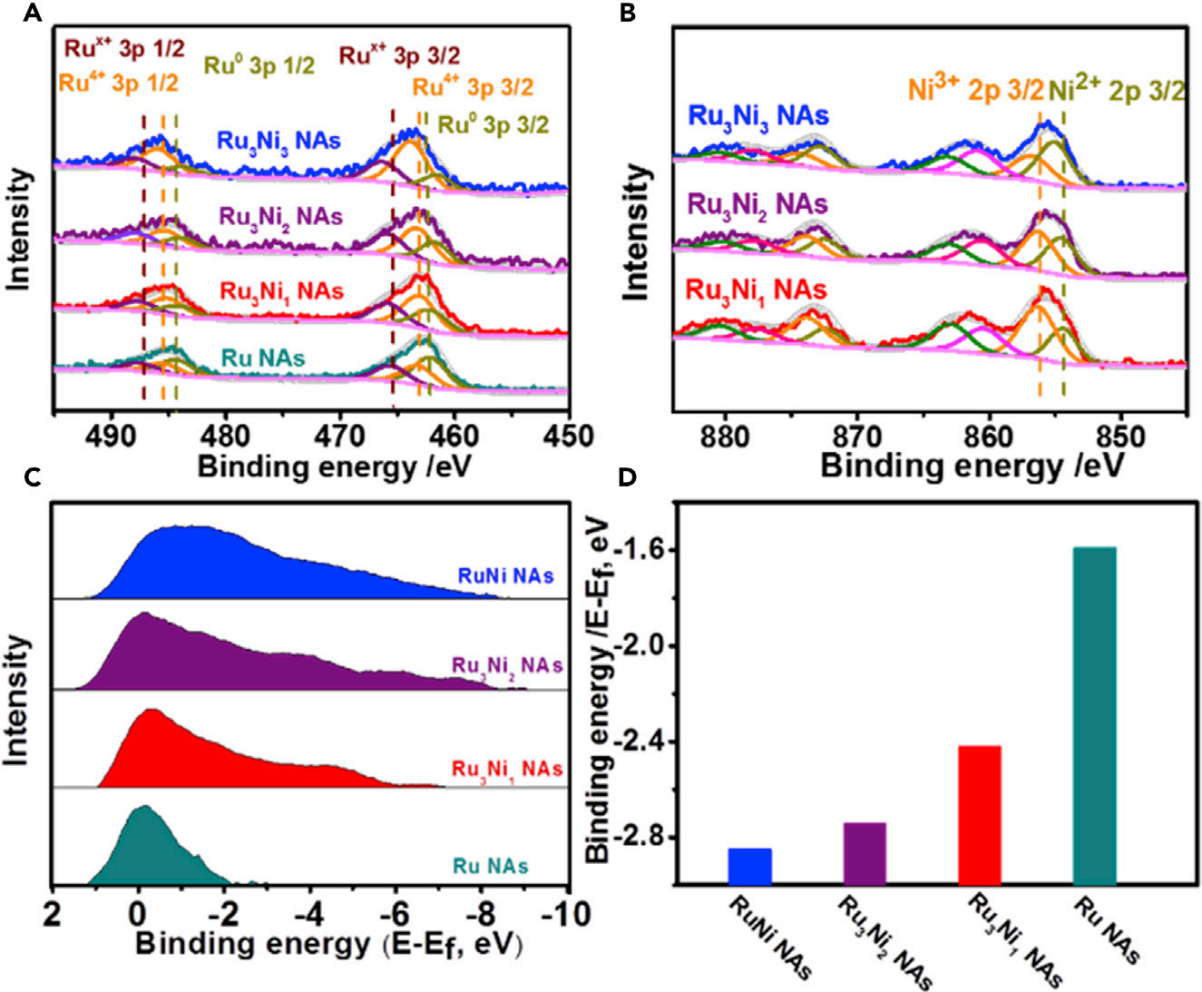
XPS Analysis of the Ru_3_Ni_3_ NAs, Ru_3_Ni_2_ NAs, Ru_3_Ni_1_ NAs, and Ru NAs (A and B) (A) Ru 3p and (B) Ni 2p curves of the Ru_3_Ni_3_ NAs, Ru_3_Ni_2_ NAs, Ru_3_Ni_1_ NAs, and Ru NAs heated at 350°C in air for 2 h. (C and D) (C) Surface valence band photoemission spectra and (D) corresponding d-band centers of the Ru_3_Ni_3_ NAs, Ru_3_Ni_2_ NAs, Ru_3_Ni_1_ NAs, and Ru NAs heated at 250°C in air for 1 h.

It should be noted that both the HER and OER activities of the Ru-Ni NAs in different electrolytes are higher than those of most catalysts reported to date (Tables S1–S3). To explore the reasons behind the high performance, the surface structures of the different catalysts were first explored in detail. As shown in Figure S10, no obvious morphological changes were found in the Ru-Ni NAs after heat treatment. However, the XRD peaks assigned to RuO_2_ appeared in the catalysts processed at 350°C in air, and the Ru_3_Ni_3_ NAs showed the highest peak for RuO_2_ (Figure S21A). Considering that RuO_2_ plays an important role in enhancing the OER activity, the formed RuO_2_ greatly enhances the OER activity in the Ru-Ni NAs (Fang and Liu, 2010). XPS was also carried out to explore the surface properties of the Ru-Ni NAs. Figure S22 shows the full scan curves of the different Ru-Ni NAs, and the positions of the Ru and Ni peaks were consistent with the literature results (Folkesson et al., 1973). Furthermore, the XPS peaks of Ru in different catalysts after treatment at 350°C in air for 2 h were divided into Ru 3p3/2 and Ru 3p1/2 peaks, which can be further split into three peaks, corresponding to Ru^x+^ (purple line), Ru^4+^ (orange line), and Ru^0^ (dark yellow line) (Figure 5A) (Li et al., 2016). It was calculated that the Ru^4+^ fractions in the Ru_3_Ni_3_ NAs (57.00%), Ru_3_Ni_2_ NAs (44.46%), and Ru_3_Ni_1_ NAs (44.57%) were much higher than those in the Ru NAs (29.89%) (Table S4), which confirmed the higher concentrations of RuO_2_ in the Ru-Ni NAs. As shown in Figure 5B, the Ni 2p peaks in the Ru_3_Ni_3_ NAs, Ru_3_Ni_2_ NAs, and Ru_3_Ni_1_ NAs were composed of Ni 2p1/2 and Ni 2p3/2 peaks, which both split into two oxidized Ni peaks, namely, Ni^2+^ (dark yellow line) and Ni^3+^ (orange line) (Zhang et al., 2007, Gong and Dai, 2015). Ni^3+^ is helpful for the formation of NiOOH on the catalyst surface, resulting in a better OER performance (Lee et al., 2012). This result indicates that both Ru and Ni in high oxidation states are generated in the Ru-Ni NAs by treatment at 350°C for 2 h in air, and that they are beneficial for the enhanced OER performance.

Compared with the peaks of Ru-Ni NAs treated at 350°C for 2 h in air, no additional peaks were generated for the Ru-Ni NAs treated at 250°C for 1 h in air (Figure S21B). Based on XPS analysis, Ru can be successfully split into three peaks, namely, Ru^x+^ (purple line), Ru^4+^ (orange line), and Ru^0^ (dark yellow line) (Figure S23). It was calculated that the area ratios of the metallic Ru0 were 59.74%, 56.77%, and 58.06% in the Ru_3_Ni_3_ NAs, Ru_3_Ni_2_ NAs, and Ru_3_Ni_1_ NAs, respectively, which were higher than 56.08% in the Ru NAs. (Table S5) and indicated a large number of active sites of metallic Ru present on the surface of the Ru_3_Ni_3_ NAs. Surface valence band XPS spectra were also obtained to determine the d-band centers of the Ru-Ni NAs treated at 250°C in air (Figures 5C and 5D). The d-band center downshifted with the increasing concentration of Ni. The reported d-band centers of Pt and Ru are located at −2.32 and −1.49 eV, respectively, corresponding to hydrogen binding energies of −0.32 and −0.64 eV, respectively, and suggesting that Ru shows a stronger hydrogen adsorption than Pt (Jiao et al., 2015). Pt is regarded as the best catalyst for HER performance owing to the suitable binding energy between the catalysts and adsorbates. Here, by alloying the catalyst with Ni, a downshift of the d-band center was observed in the Ru-Ni NAs (Figures 5C and 5D), which results in a suitable binding energy between the Ru-Ni NAs and adsorbates and boosted the HER activity of the Ru-Ni NAs (Stamenkovic et al., 2007).

**Figure 6:**
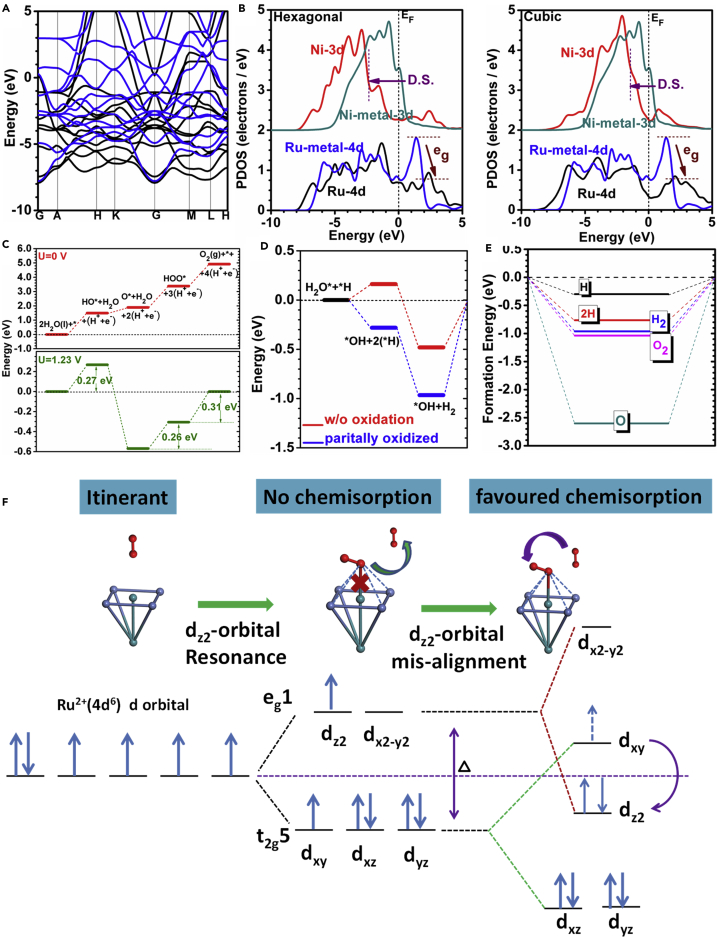
DFT Simulations of OER and HER (A) The simulated band structure for the Ru-Ni hexagonal alloy. (B) The PDOS of Ru-Ni. (C) Free energy path for the OER. (D) Free energy path for alkaline HER. (E) The chemisorption energy for the acidic HER and neutral OER. (F) Schematic illustration of the transition of electrons from t_2g_ to e_g_ orbital and the evolution of intermediate spin state to high spin state on Ru-Ni (100) surface. Green ball, Ru; blue ball, Ni; red ball, oxygen molecule.

We further carried out density functional theory (DFT) calculations to elucidate how the downshift effect of the Ru-Ni NAs is related to the high performance of water splitting for both the OER and HER. The Ru-Ni NA system was modeled by a hexagonal lattice (hex-Ru-Ni) based on the Ru local symmetry. It shows a good metallic behavior with uniform isotropic conductivity across the Fermi level (E_F_) (Figure 6A). The d-orbital projected density of states (PDOS) were compared and showed that Ni-3d downshifted to a value lower than that obtained for the bulk face-centered cubic Ni metal (Figure 6B) due to repulsion with the overlapping Ru-4d orbital, which implied a weakening in the Ni-O and Ni-H bonding. In addition, this downshifting effect appeared to be even more pronounced within the hexagonal local lattice than in the cubic lattice. Meanwhile, the Ru-4d states also downshifted compared with those in the hex-Ru metal, especially for the 4d-e_g_ component above the E_F_ (Figure 6B), regardless of the different local symmetries. This occurs because the eg-level component is essential for the adsorption of the bond of the p-π lone pair electrons in molecules such as H_2_O, O, or O_2_. This is because they almost remain in the non-bonding orbitals, and the adsorption stabilities are dominated by the Coulomb repulsion between 4d-e_g_ in such p-π orbitals. Accordingly, the Ru in hex-Ru-Ni will easily transfer electrons between the catalysis substrate and intermediate molecules and facilitate O-O bond formation. The simulated OER pathway (Figure 6C) shows that the system is an energetically favorable catalyst even under U = 0 and U = 1.23 V, showing that water splitting with such Ru-Ni NAs would be a substantially low-barrier process. The splitting of H_2_O results in an increase in energy of 1.49 eV, guaranteeing that the initiation would be very reactive within a low overpotential. Meanwhile, there is no evident change in the energy for the evolution reaction [HO*+(H^+^+e^−^)]→[O*+2(H^+^+e^−^)] (∼0.4 eV). An additional similarly energetic increase (1.50 eV) was found for the formation of *OOH, indicating that the O* on the Ru-Ni still stays active to oxidize OH under lower overpotential. The splitting of H for the [HOO*+3(H^+^+e^−^)]→[O_2_+4(H^+^+e^−^)] transformation is very active. Compared with the pathway at U = 1.23 V, we confirm the overall overpotential (i.e., η = max{[barrier-1.23 eV]/e = 0.306 V}) is almost the same within the range of 0.200–0.300 V. Further calculations of the O_2_ dissociation confirmed that the combined O-O on the Ru-contained surface will be easily dissociated and enter into the surrounding solution conditions (Figure S24, Tables S6 and S7). Therefore, the OER on the Ru-Ni surface can achieve a very high performance supported by an energetic barrier-free water-splitting process. We further gain energetic insights on the alkaline HER. In the Ru-Ni surface system without partial oxidations by O-coverage, the alkaline HER performance overall is energetically downhill and the whole process gains a reaction heat of −0.48 eV with a small barrier of 0.16 eV. Activation barrier for the HER on this system may arise due to barrier of [H_2_O→H + OH]. As found by our experimental observation, partial oxidation states were found on the surface. We further conducted the reaction energy calculation. The overall reaction heat released is found to be −0.97 eV, showing it to be rather more energetically favorable than the case without oxidation. The process of [H_2_O→H + OH] is also energetically preferred gaining −0.28 eV during the bond cleavage on the partially oxidized Ru-Ni surface (Figure 6D). At the same time, a comparison of the chemisorption energies sheds light on the high HER/OER performance (Figure 6E). We also determined that the HER on the Ru-Ni system favors high H coverage with easy chemisorption of the 2H, and the formation of 2H→H_2_ is energetically favorable. Meanwhile, the low O coverage will easily facilitate water splitting and further accelerate further 2O chemisorption and O_2_ desorption. The kinetics of possible oxygen absorption or oxygen-related intermediates (OH^−^) is shown in absorption process in Figure 6F, which will result in the formation an intermediate distorted octahedral unit. The overlapping between eg orbital of Ru^2+^ and O-p_σ_ orbitals will facilitate the ion transfer. The distorted structure prompts Ru^2+^ (d^6^) to change from a low-spin state (t_2g_^6^e_g_^0^) to an intermediate-spin state (t_2g_^5^e_g_^1^), where the eg^1^ can point to the intermediate with high bonding possibility. We also find that the absorption energy of further absorption on vertical oxygen molecule will be lowered nearly 1 eV, which can be attributed to the Jahn-Teller effect from the extra oxygen molecule to the c-axis of the distorted octahedral unit, which decreases the whole energy. Electrons on t_2g_ can be further excited to eg and then form a high-spin state (t_2g_^4^e_g_^2^) with energy decrease. Overall, the Ru-Ni catalytic system is found to be efficient in HER performance from acidic to the basic condition. Thus, the Ru-Ni (NAs) system exhibits a high catalytic reactivity for water splitting based on the DFT calculations. We have also made a detail comparison for the preliminary absorption behavior on the cubic Ru-Ni (111) and hexagonal close packed (hcp) Ru-Ni (001) surface to elucidate the experimental treatment and related analysis. The discussions and analysis cover the following sections: energetics, electronic structures, orbital energetic behaviors, and adsorption analysis (Figures S24–S28 in Supplemental Information).

In summary, for the first time, we have demonstrated a facile method for the synthesis of 3D Ru-Ni NAs, which leads to favorable 3D Ru-Ni superstructures with fully exposed active sites. The valence band spectra and DFT calculations revealed a change in the d-band center in the Ru-Ni NAs after the introduction of Ni, resulting in the transformation to a favorable surface environment for the OER and HER. The RuO_2_-decorated Ru-Ni NAs treated at 350°C in air provided additional active sites for the OER. The combined structural and electronic engineering leads to superior electrocatalytic performance for overall water splitting under universal pH conditions, and the performance is much better than that of the commercial Pt/C and Ir/C, demonstrating an unprecedented class of nanocatalysts with exceptional activity and excellent stability for electrochemical water splitting.

### Limitations of the Study

Our work has demonstrated a novel bifunctional catalyst for water splitting in the universal environment from experimental and theoretical perspectives. Based on the combination of XPS and DFT as an effective approach, electronic environment modulation has been interpreted as the key factor that facilitates both HER and OER. However, an in-depth understanding of the oxidation states of the catalyst is still an open challenge because of the complex charge transfer induced by the overlap between the metal orbitals as well as the correspondingly accurate characterization. The site-to-site sampling and analysis of surface oxidation sites is of great significance for precise understanding of the catalyst reactivity. Therefore, we will keep working on further development and perfection on related theoretical exploration and advancement.

## Methods

All methods can be found in the accompanying Transparent Methods supplemental file.

## Supplemental Information

Document S1. Transparent Methods, Figures S1–S28, and Tables S1–S7

## References

[bib1] Balogun M.-S., Qiu W., Yang H., Fan W., Huang Y., Fang P., Li G., Ji H., Tong Y. (2016). A monolithic metal-free electrocatalyst for oxygen evolution reaction and overall water splitting. Energy Environ. Sci..

[bib2] Chen S., Liu G., Yadegari H., Wang H., Qiao S.Z. (2015). A Three-dimensional MnO_2_ ultrathin nanosheet aerogels for high-performance Li-O_2_ batteries. J. Mater. Chem..

[bib3] Cherevko S., Geiger S., Kasian O., Kulyk N., Grote J.-P., Savan A., Shrestha B.R., Merzlikin S., Breitbach B., Ludwig A., Mayrhofer K.J.J. (2016). Oxygen and hydrogen evolution reactions on Ru, RuO2, Ir, and IrO2 thin film electrodes in acidic and alkaline electrolytes: a comparative study on activity and stability. Catal. Today.

[bib4] Chhowalla M., Shin H.S., Eda G., Li L.-J., Loh K.P., Zhang H. (2013). The chemistry of two-dimensional layered transition metal dichalcogenide nanosheets. Nat. Chem..

[bib5] Conway B.E., Tilak B.V. (2002). Interfacial processes involving electrocatalytic evolution and oxidation of H_2_, and the role of chemisorbed H. Electrochim. Acta.

[bib6] Dresselhaus M.S., Thomas I.L. (2001). Alternative energy technologies. Nature.

[bib7] Ellis W.C., McDaniel N.D., Bernhard S., Collins T.J. (2010). Fast water oxidation using iron. J. Am. Chem. Soc..

[bib8] Fang Y.-H., Liu Z.-P. (2010). Mechanism and Tafel lines of electro-oxidation of water to oxygen on RuO_2_ (110). J. Am. Chem. Soc..

[bib9] Folkesson B., Bjorøy M., Pappas J., Skaarup S., Aaltonen R., Swahn C.G. (1973). ESCA studies on the charge distribution in some dinitrogen complexes of rhenium, iridium, ruthenium, and osmium. Acta Chem. Scand..

[bib10] Gao Q., Giordano C., Antonietti M. (2012). Biomimetic oxygen activation by MoS_2_/Ta_3_N_5_ nanocomposites for selective aerobic oxidation. Angew. Chem. Int. Ed..

[bib11] Gong M., Dai H. (2015). A mini review of NiFe-based materials as highly active oxygen evolution reaction electrocatalysts. Nano Res..

[bib12] Hang L., Wu H.B., Yan Y., Wang X., Lou X.W. (2014). Hierarchical MoS_2_ microboxes constructed by nanosheets with enhanced electrochemical properties for lithium storage and water splitting. Energy Environ. Sci..

[bib13] Hwang H., Kim H., Cho J. (2011). MoS_2_ nanoplates consisting of disordered graphene-like layers for high rate lithium battery anode materials. Nano Lett..

[bib14] Jiang Y., Li X., Yu S., Jia L., Zhao X., Wang C. (2015). Reduced graphene oxide-modified carbon nanotube/polyimide film supported MoS_2_ nanoparticles for electrocatalytic hydrogen evolution. Adv. Funct. Mater..

[bib15] Jiao Y., Zheng Y., Jaroniec M., Qiao S.Z. (2015). Design of electrocatalysts for oxygen-and hydrogen-involving energy conversion reactions. Chem. Soc. Rev..

[bib16] Jin H., Wang J., Su D., Wei Z., Pang Z., Wang Y. (2015). In situ cobalt-cobalt oxide/N-vdoped carbon hybrids as superior bifunctional electrocatalysts for hydrogen and oxygen evolution. J. Am. Chem. Soc..

[bib17] Jin Y., Wang H., Li J., Yue X., Han Y., Shen P.K., Cui Y. (2016). Porous MoO_2_ nanosheets as non-noble bifunctional electrocatalysts for overall water splitting. Adv. Mater..

[bib18] Kong X., Xu K., Zhang C., Dai J., Norooz Oliaee S., Li L., Zeng X., Wu C., Peng Z. (2016). Free-standing two-dimensional Ru nanosheets with high activity toward water splitting. ACS Catal..

[bib19] Lee Y., Suntivich J., May K.J., Perry E.E., Shao-Horn Y. (2012). Synthesis and activities of rutile IrO_2_ and RuO_2_ nanoparticles for oxygen evolution in acid and alkaline solutions. J. Phys. Chem. Lett..

[bib20] Lettenmeier P., Wang L., Golla-Schindler U., Gazdzicki P., Cañas N.A., Handl M., Hiesgen R., Hosseiny S.S., Gago A.S., Friedrich K.A. (2016). Nanosized IrO_x_-Ir catalyst with relevant activity for anodes of proton exchange membrane electrolysis produced by a cost-effective procedure. Angew. Chem. Int. Ed..

[bib21] Li X., Liu J., Huang Q., Vogel W., Akins D.L., Yang H. (2016). Effect of heat treatment on stability of gold particle modified carbon supported Pt-Ru anode catalysts for a direct methanol fuel cell. Electrochim. Acta.

[bib22] Lu X.Y., Zhao C.E. (2015). Electrodeposition of hierarchically structured three-dimensional nickel-iron electrodes for efficient oxygen evolution at high current densities. Nat. Commun..

[bib23] Lu Z.Y., Wang H.T., Kong D.S., Yan K., Hsu P.C., Zheng G.Y., Yao H.B., Liang Z., Sun X.M., Cui Y. (2014). Electrochemical tuning of layered lithium transition metal oxides for improvement of oxygen evolution reaction. Nat. Commun..

[bib24] Ma Y.-Y., Wu C.-X., Feng X.-J., Tan H.-Q., Yan L.-K., Liu Y., Kang Z.-H., Wang E.-B., Li Y.-G. (2017). Highly efficient hydrogen evolution from seawater by a low-cost and stable CoMoP@ C electrocatalyst superior to Pt/C. Energy Environ. Sci..

[bib25] Mahmood J., Li F., Jung S.-M., Okyay M.S., Ahmad I., Kim S.-J., Park N., Jeong H.Y., Baek J.-B. (2017). An efficient and pH-universal ruthenium-based catalyst for the hydrogen evolution reaction. Nat. Nanotechnol..

[bib26] Petrykin V., Macounova K., Shlyakhtin O.A., Krtil P. (2010). Tailoring the selectivity for electrocatalytic oxygen evolution on ruthenium oxides by zinc substitution. Angew. Chem. Int. Ed..

[bib27] Pu Z., Amiinu I.S., Kou Z., Li W., Mu S. (2017). RuP_2_-based catalysts with platinum-like activity and higher durability for hydrogen evolution reaction at all pH value. Angew. Chem. Int. Ed..

[bib28] Reier T., Oezaslan M., Strasser P. (2012). Electrocatalytic oxygen evolution reaction (OER) on Ru, Ir, and Pt catalysts: a comparative study of nanoparticles and bulk materials. ACS Catal..

[bib29] Seitz L.C., Dickens C.F., Nishio K., Hikita Y., Montoya J., Doyle A., Kirk C., Vojvodic A., Hwang H.Y., Norskov J.K., Jaramillo T.F. (2016). A highly active and stable IrOx/SrIrO_3_ catalyst for the oxygen evolution reaction. Science.

[bib30] Stamenkovic V.R., Fowler B., Mun B.S., Wang G., Ross P.N., Lucas C.A., Marković N.M. (2007). Improved oxygen reduction activity on Pt_3_Ni (111) via increased surface site availability. Science.

[bib31] Turner J.A. (2004). Sustainable hydrogen production. Science.

[bib32] Wang X., Zhuang L., He T., Jia Y., Zhang L., Yan X., Gao M., Du A., Zhu Z., Yao X., Yu S.-H. (2018). Grafting cobalt diselenide on defective graphene for enhanced oxygen evolution reaction. iScience.

[bib33] Wang X., Zhuang L., Jia Y., Liu H., Yan X., Zhang L., Yang D., Zhu Z., Yao X. (2018). Plasma-triggered synergy of exfoliation, phase transformation, and surface engineering in cobalt diselenide for enhanced water oxidation. Angew. Chem. Int. Ed..

[bib34] Zhang J., Wang H., Dalai A.K. (2007). Development of stable bimetallic catalysts for carbon dioxide reforming of methane. J. Catal..

[bib35] Zhang J., Wang T., Liu P., Liao Z., Liu S., Zhuang X., Chen M., Zschech E., Feng X. (2017). Efficient hydrogen production on MoN_i4_ electrocatalysts with fast water dissociation kinetics. Nat. Commun..

[bib36] Zhang N., Shao Q., Pi Y., Guo J., Huang X. (2017). Solvent-mediated shape tuning of well-defined rhodium nanocrystals for efficient electrochemical water splitting. Chem. Mater..

[bib37] Zhang Y., Xia X., Cao X., Zhang B., Tiep N.H., He H., Chen S., Huang Y., Fan H.J. (2017). Ultrafine metal nanoparticles/N-doped porous carbon hybrids coated on carbon fibers as flexible and binder-free water splitting catalysts. Adv. Energy Mater..

[bib38] Zheng Y., Jiao Y., Zhu Y., Li L.H., Han Y., Chen Y., Du A., Jaroniec M., Qiao S.Z. (2014). Hydrogen evolution by a metal-free electrocatalyst. Nat. Commun..

[bib39] Zheng Y., Jiao Y., Zhu Y., Li L.H., Han Y., Chen Y., Jaroniec M., Qiao S.-Z. (2016). High electrocatalytic hydrogen evolution activity of an anomalous ruthenium catalyst. J. Am. Chem. Soc..

