# Supplementary material for: pH-Universal Water Splitting Catalyst: Ru-Ni Nanosheet Assemblies
Source: iScience. 2019 Jan 5;11:492–504. doi: 10.1016/j.isci.2019.01.004 (PMC6348166; doi:10.1016/j.isci.2019.01.004)
Supplement: Document S1. Transparent Methods, Figures S1–S28, and Tables S1–S7 [file mmc1.pdf]

ISCI, Volume *11*

Supplemental Information

pH-Universal Water Splitting Catalyst: Ru-Ni Nanosheet Assemblies

Jian Yang, Qi Shao, Bolong Huang, Mingzi Sun, and Xiaoqing Huang

Supporting Information

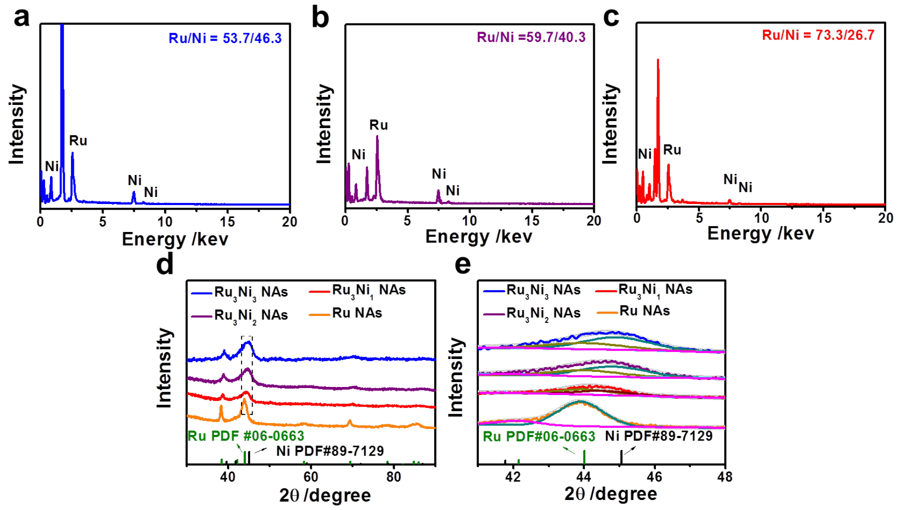

Figure S1. EDS spectra and XRD characterization of synthesized RuNi NAs, related to Figure 1.

(a-c) SEM-EDS spectra and (d) XRD patterns of Ru_3Ni_3 NAs, Ru_3Ni_2 NAs, Ru_3Ni_1 NAs and Ru NAs. (e) The main peak enlarged drawing corresponding to (d).

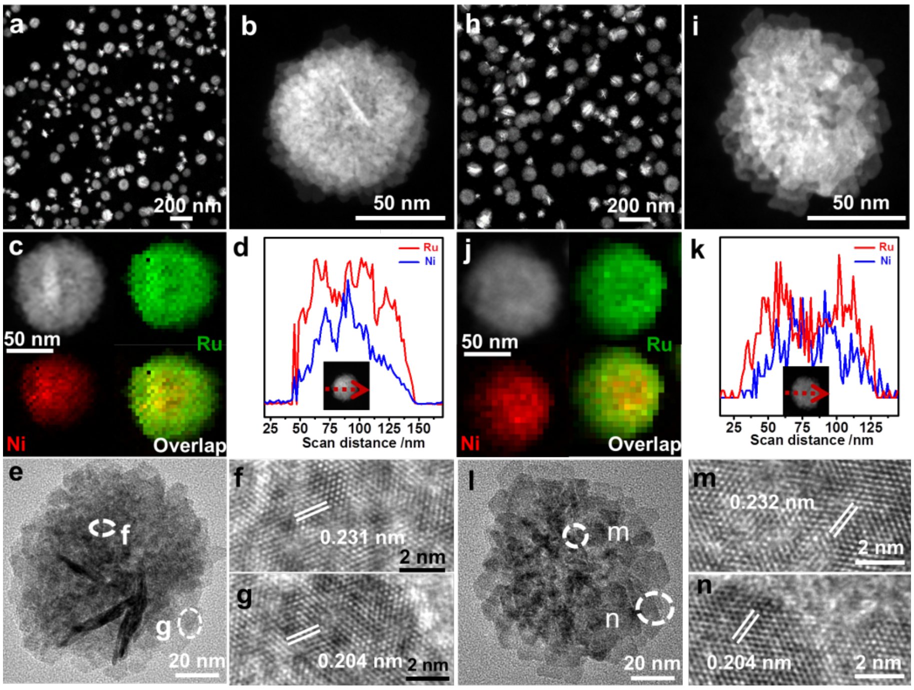

Figure S2. STEM, EDS and TEM images of synthesized RuNi NAs, related to Figure 1.

(a, b, h, i) HAADF-STEM images, (c, j) EDS elemental mapping images and (d, k) line scans of (a-d) Ru_3Ni_2 NA and (h-k) Ru_3Ni_1 NA. (e, l) TEM images and (f, g, m, n) HRTEM images of (e-g) Ru_3Ni_2 NA and (l-n) Ru_3Ni_1 NA.

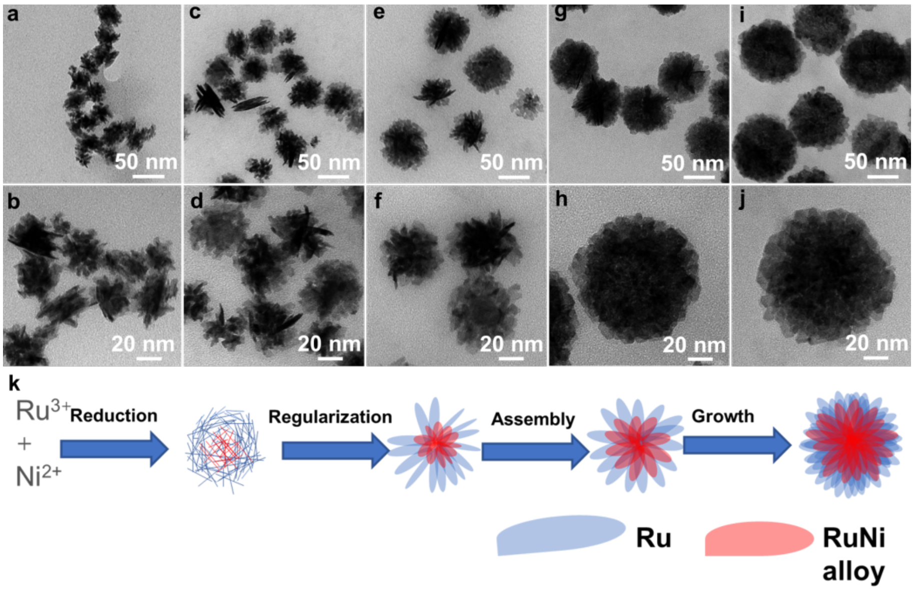

Figure S3. TEM images of the intermediates of the Ru_3Ni_3 NAs obtained at different reaction times, related to Figure 1.

(a, b) t = 25 min, (c, d) t = 40 min, (e, f) 1.5 h, (g, h) t = 3 h and (i, j) t = 5 h. (k) Schematic illustration on the growth of the Ru_3Ni_3 NAs.

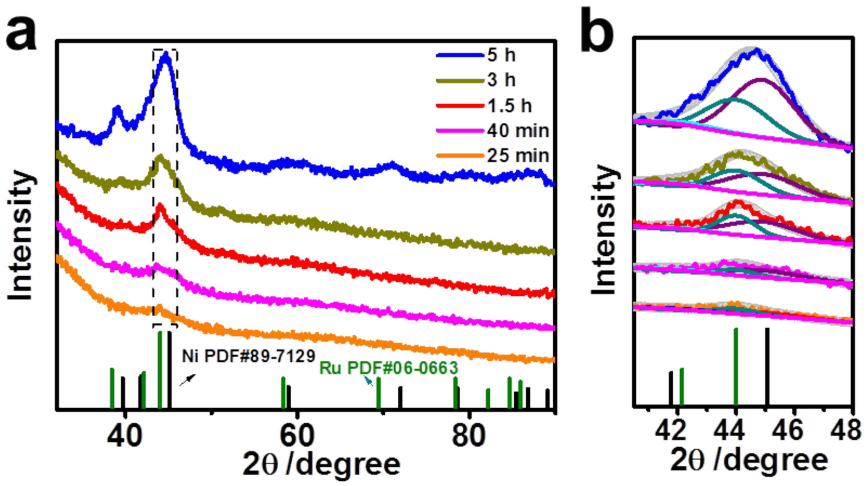

Figure S4. XRD characterization of the Ru_3Ni_3 NAs, related to Figure 1. (a) XRD patterns of the intermediates of the Ru_3Ni_3 NAs obtained at different reaction time. (b) The main peak enlarged from the dashed region marked in (a).

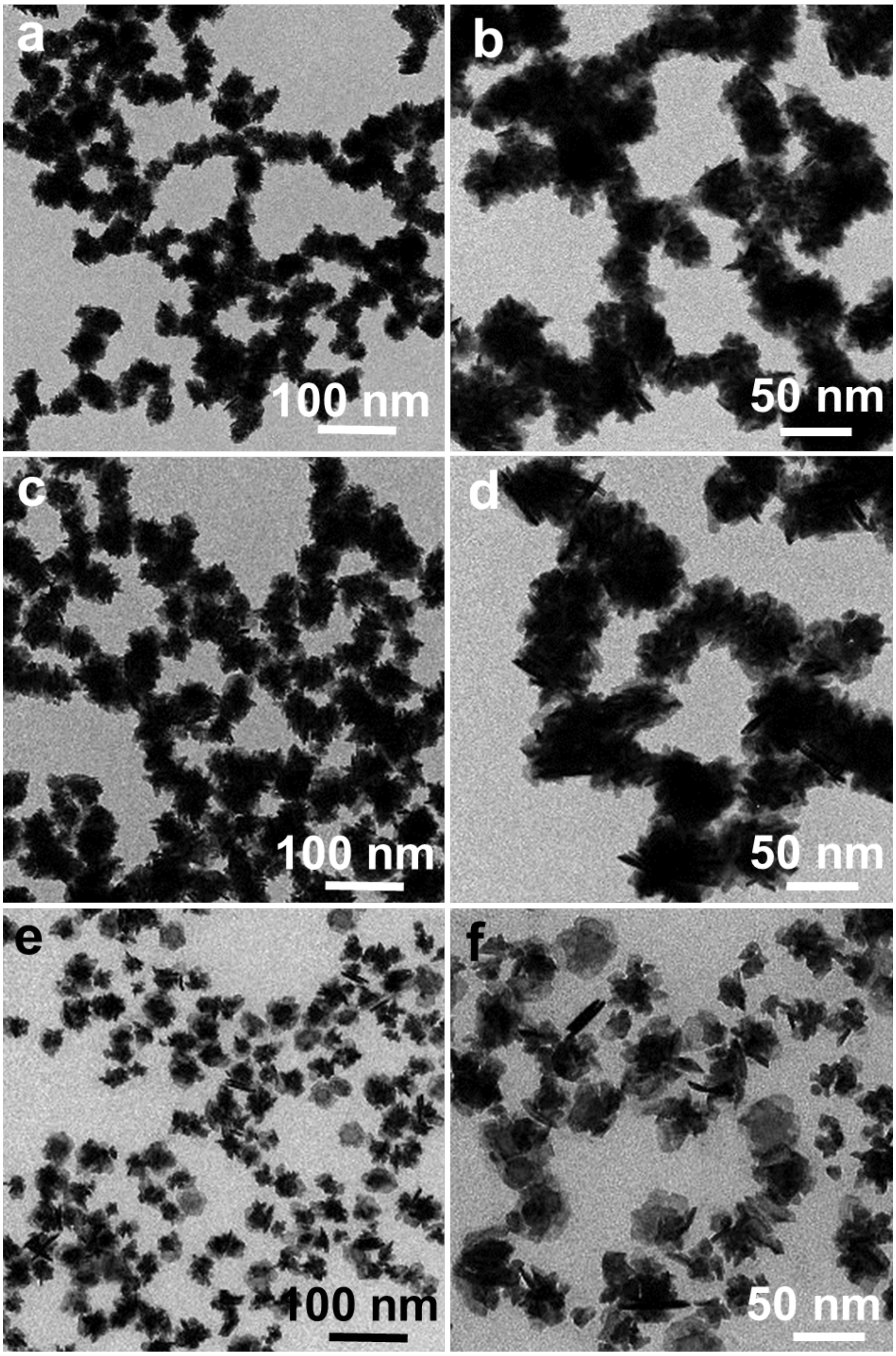

Figure S5. TEM images of the obtained products using the standard procedure for the Ru_3Ni_3 NAs except for using different amounts of phloroglucinol, related to Figure 1.
(a, b) 0 mg (c, d) 25.3 mg and (e, f) 100 mg.

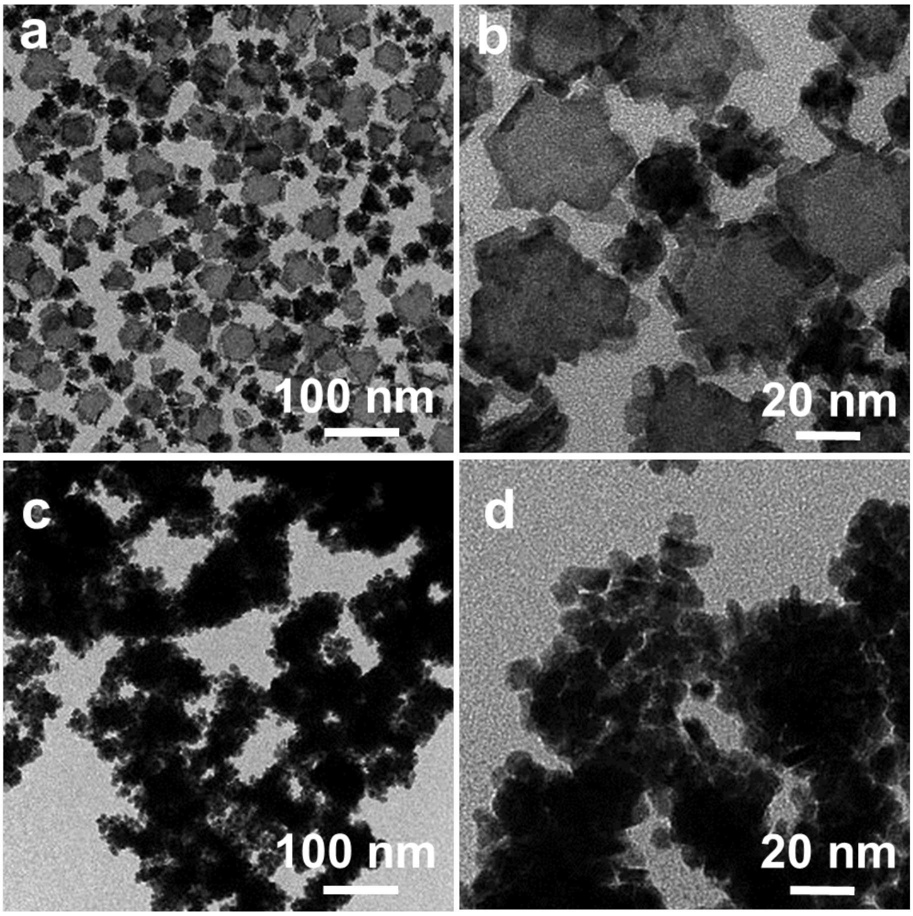

Figure S6. TEM images of the obtained products using the standard procedure for the Ru_3Ni_3 NAs except for using different amounts of tetramethylammonium bromide, related to Figure 1.
(a, b) 0 mg and (c, d) 20 mg.

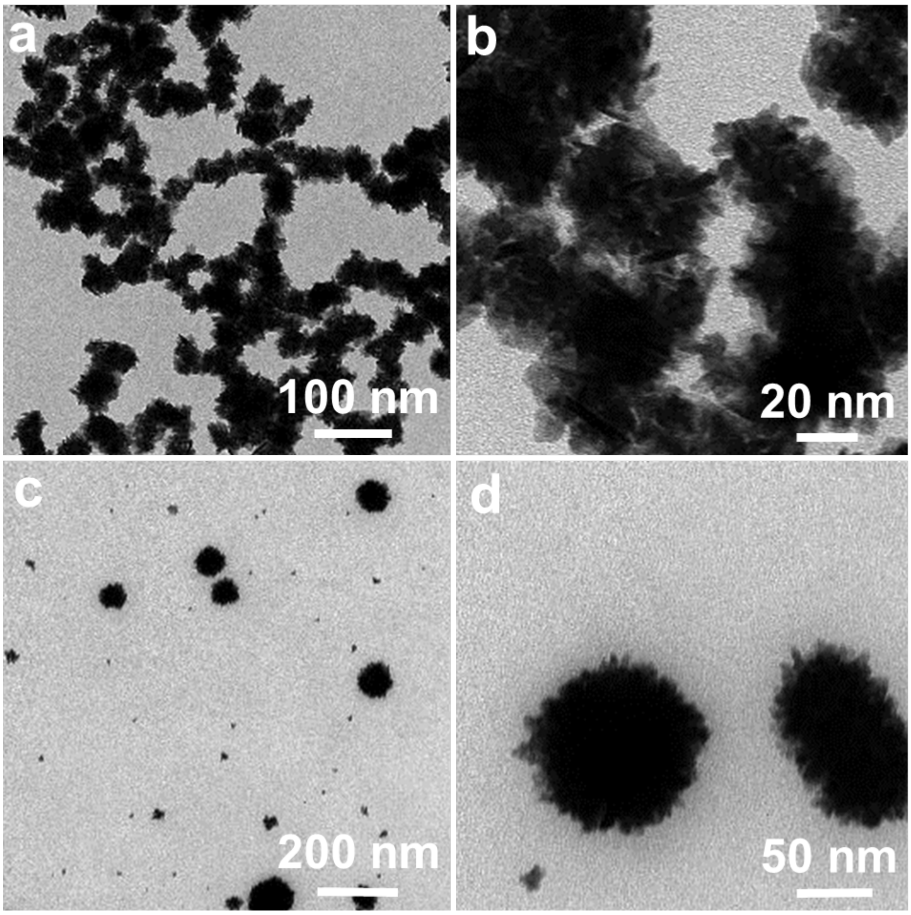

Figure S7. TEM images of the obtained products using the standard procedure for the Ru_3Ni_3 NAs, related to Figure 1.
(a, b) without using PVP and (c, d) replacing benzyl alcohol with ethylene glycol.

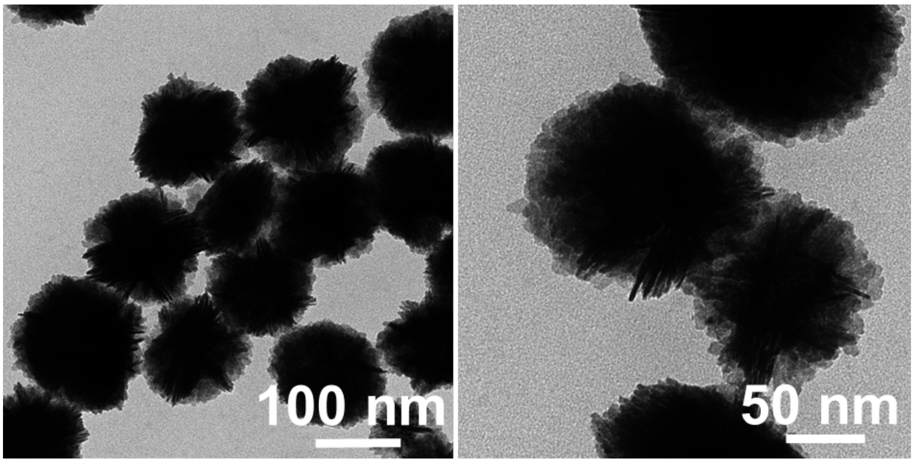

Figure S8. TEM images of Ru NAs using the standard procedure for the Ru_3Ni_3 NAs but without using $Ni(acac)_2$, related to Figure 1.

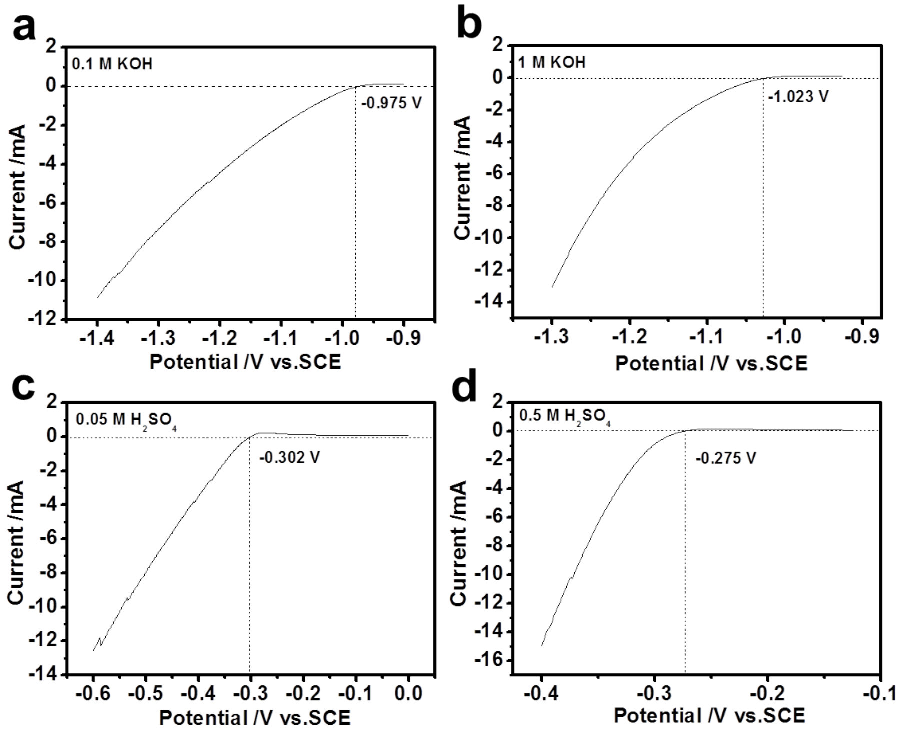

Figure S9. Calibration of the saturated calomel electrode (SCE) electrode with respect to RHE in different electrolytes. Scan rate: 1 mV s^{-1}, related to Figure 2.

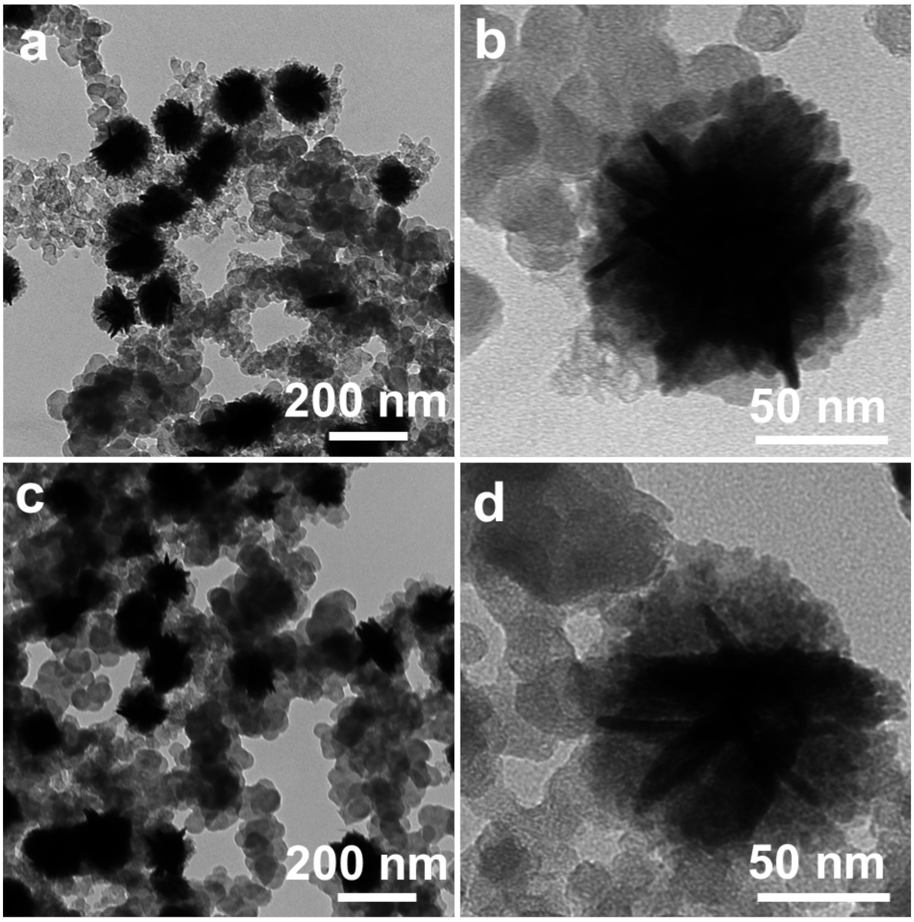

Figure S10. TEM images of Ru_3Ni_3 NAs loaded on the carbon powder after heat treatment in air at (a, b) 250 °C for 1 h and (c, d) 350 °C for 2 h, related to Figure 2.

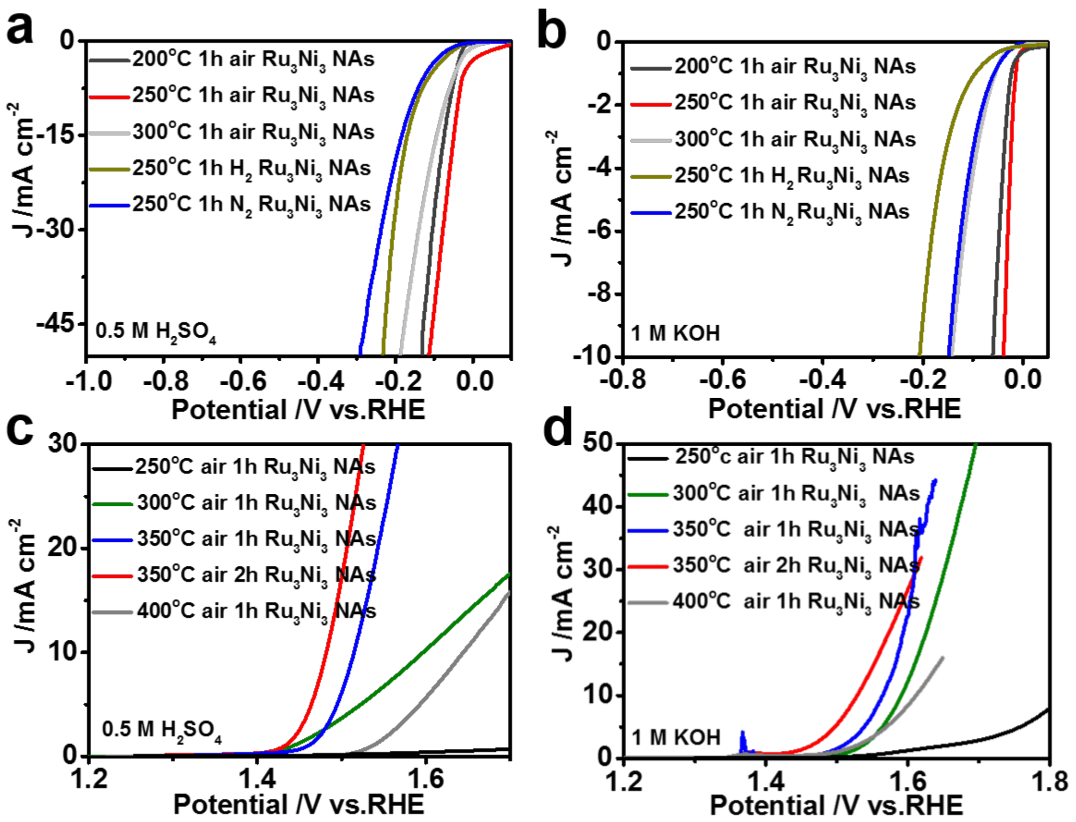

Figure S11. HER and OER performances of Ru_3Ni_3 NAs in different environment, related to Figure 2.

HER performances of Ru_3Ni_3 NAs in (a) 0.5 M H_2SO_4 and (b) 1 M KOH through heat treatment at different conditions. OER performances of Ru_3Ni_3 NAs in (c) 0.5 M H_2SO_4 and (d) 1 M KOH after heat treatment under different conditions.

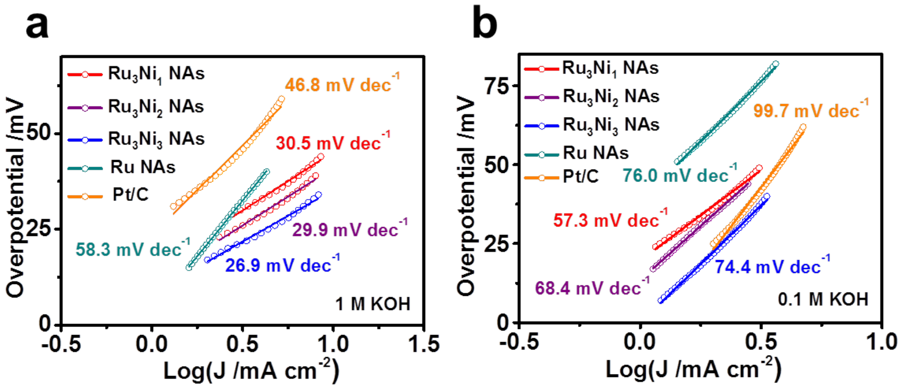

Figure S12. The corresponding HER Tafel plots of Ru_3Ni_3 NAs, Ru_3Ni_2 NAs, Ru_3Ni_1 NAs, Ru NAs and Pt/C in different environment, related to Figure 2.

(a) 1 M KOH and (b) 0.1 M KOH derived from Figure 2a, b.

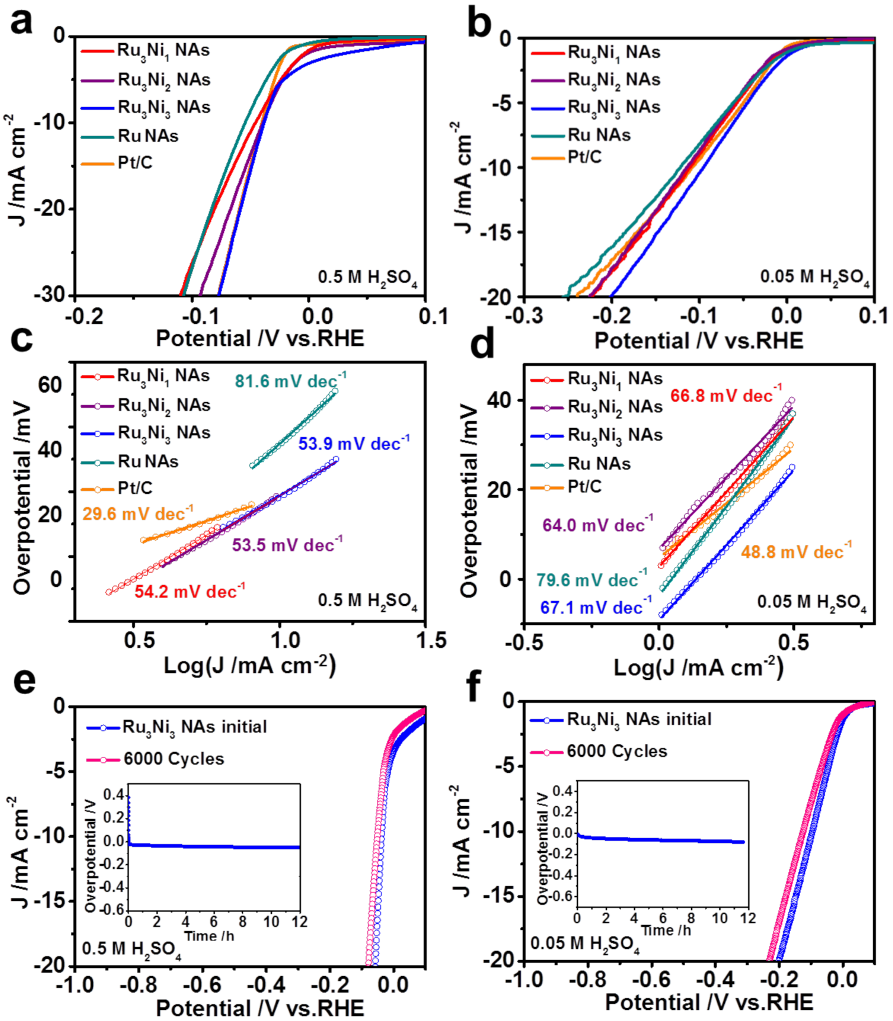

Figure S13. HER performances of Ru_3Ni_3 NAs, Ru_3Ni_2 NAs, Ru_3Ni_1 NAs, Ru NAs and Pt/C in acidic condition, related to Figure 2.

(a) The polarization curves and (c) the corresponding Tafel plots in 0.5 M H_2SO_4. (b) The polarization curves and (d) the corresponding Tafel plots in 0.05 M H_2SO_4. Scan rates are 5 mV s^{-1}. Polarization curves of Ru_3Ni_3 NAs before and after 6000 cycles in (e) 0.5 M H_2SO_4 and (f) 0.05 M H_2SO_4 at a scan rate of 5 mV s^{-1}. Inset: Chronopotentiometry curves of Ru_3Ni_3 NAs in (e) 0.5 M H_2SO_4 and (f) 0.05 M H_2SO_4 solutions at current density of 5 mA cm^{-2}.

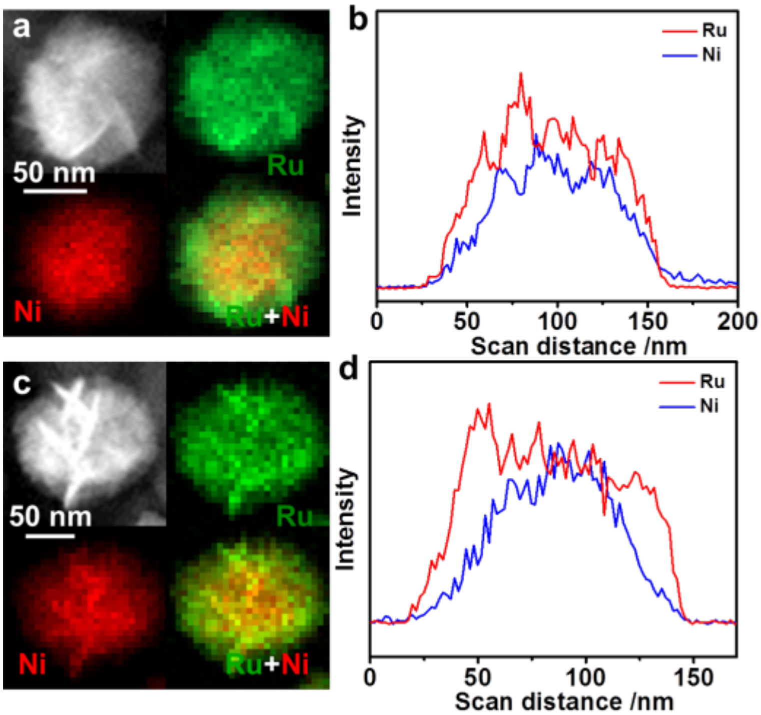

Figure S14. STEM image and elemental mapping of Ru_3Ni_3 NAs loaded on the carbon powder after heat treatment in air at different temperature and time, related to Figure 3.

(a) 250 °C for 1 h and (c) 350 °C for 2 h. Line scans of Ru_3Ni_3 NAs after heat treatment in air at (b) 250 °C for 1 h and (d) 350 °C for 2 h.

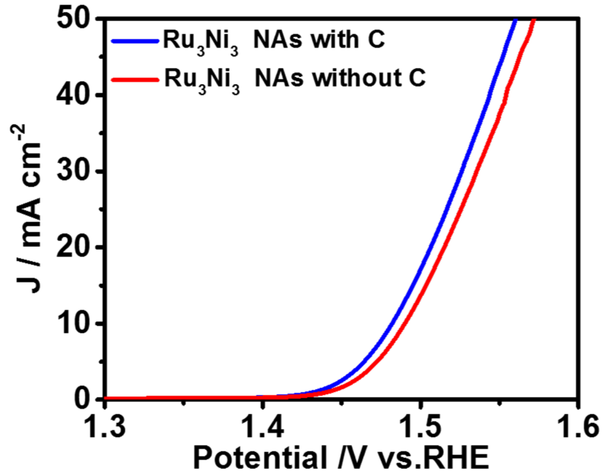

Figure S15. OER performances of Ru_3Ni_3 NAs with C and without C in the 0.5 M H_2SO_4, related to Figure 3.

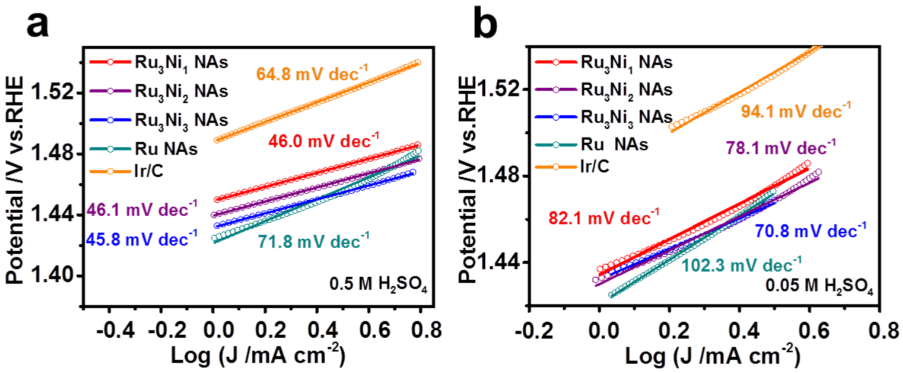

Figure S16. The corresponding OER Tafel plots of Ru_3Ni_3 NAs, Ru_3Ni_2 NAs, Ru_3Ni_1 NAs, Ru NAs and Ir/C in different acid environment, related to Figure 3.

(a) 0.5 M H_2SO_4 and (b) 0.05 M H_2SO_4 derived from Figure 3a, b.

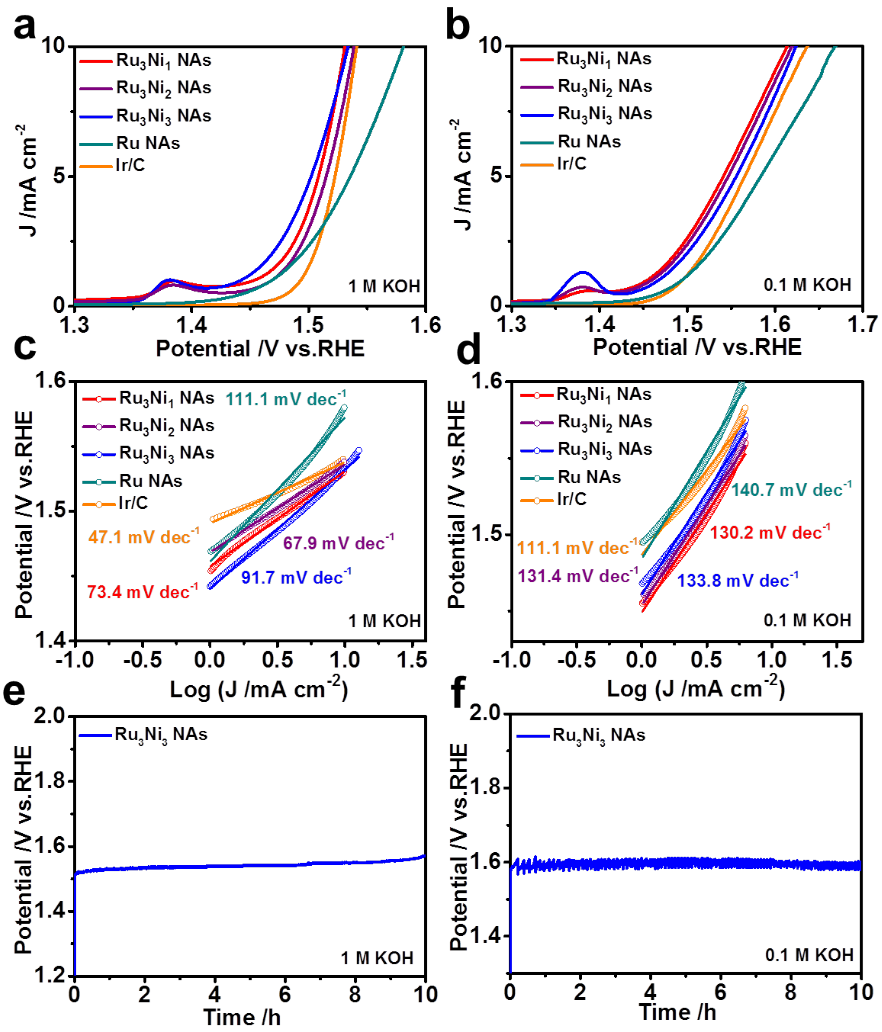

Figure S17. OER performances of Ru_3Ni_3 NAs, Ru_3Ni_2 NAs, Ru_3Ni_1 NAs, Ru NAs and Ir/C in alkaline condition, related to Figure 3.

(a) The polarization curves and (c) the corresponding Tafel plots in 1 M KOH. (b) The polarization curves and (d) the corresponding Tafel plots in 0.1 M KOH. Scan rates are 5 mV s^{-1}. Chronopotentiometry curves of Ru_3Ni_3 NAs in (e) 1 M KOH and (f) 0.1 M KOH solutions at current density of 5 mA cm^{-2}.

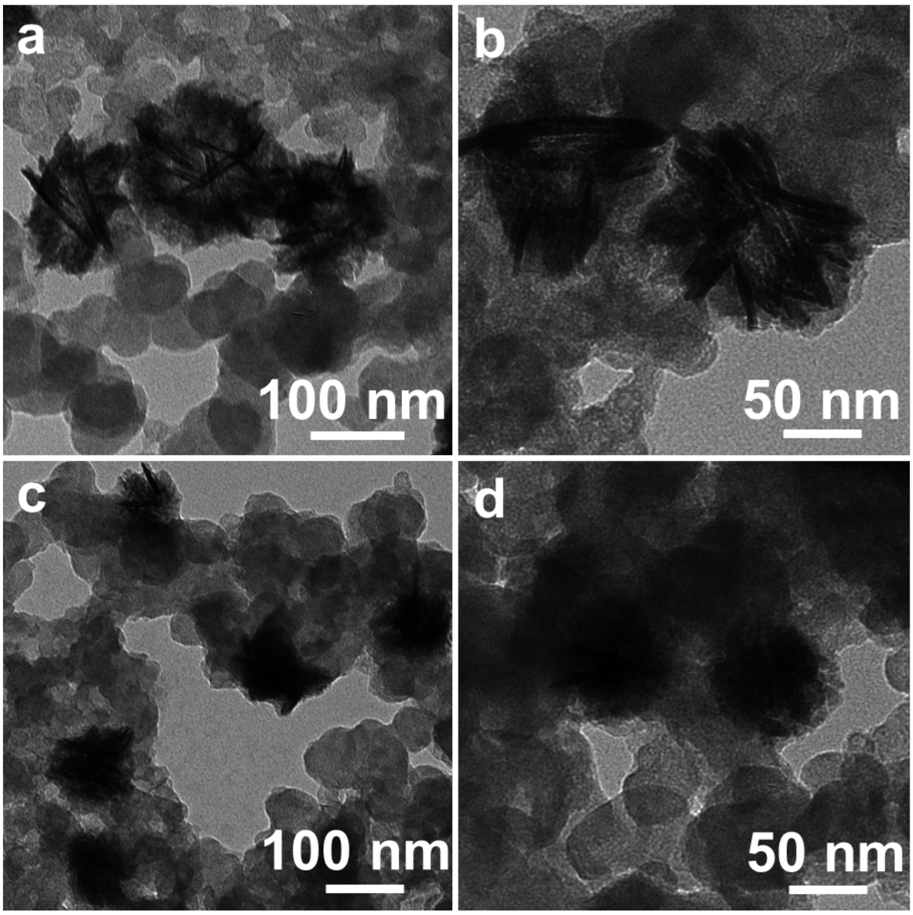

Figure S18. TEM images of Ru_3Ni_3 NAs after the OER stability tests, related to Figure 3.
(a-b) 0.5 M H_2SO_4 and (c-d) 1 M KOH.

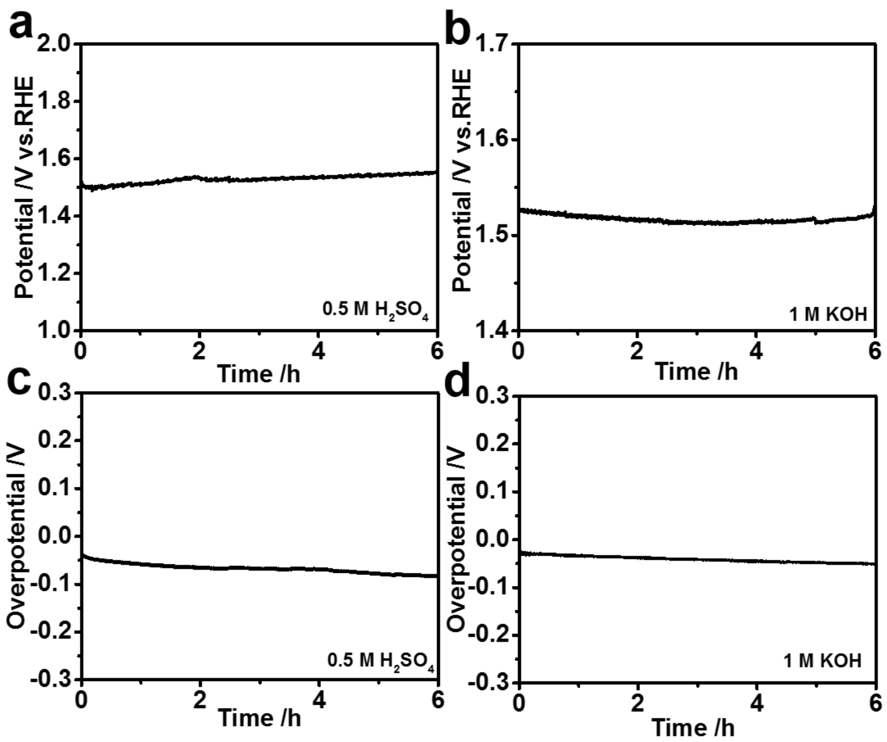

Figure S19. Chronopotentiometry curves of Ru_3Ni_3 NAs, related to Figure 3.

(a) 0.5 M H_2SO_4 and (b) 1 M KOH for OER and in (c) 0.5 M H_2SO_4 and (d) 1 M KOH for HER.

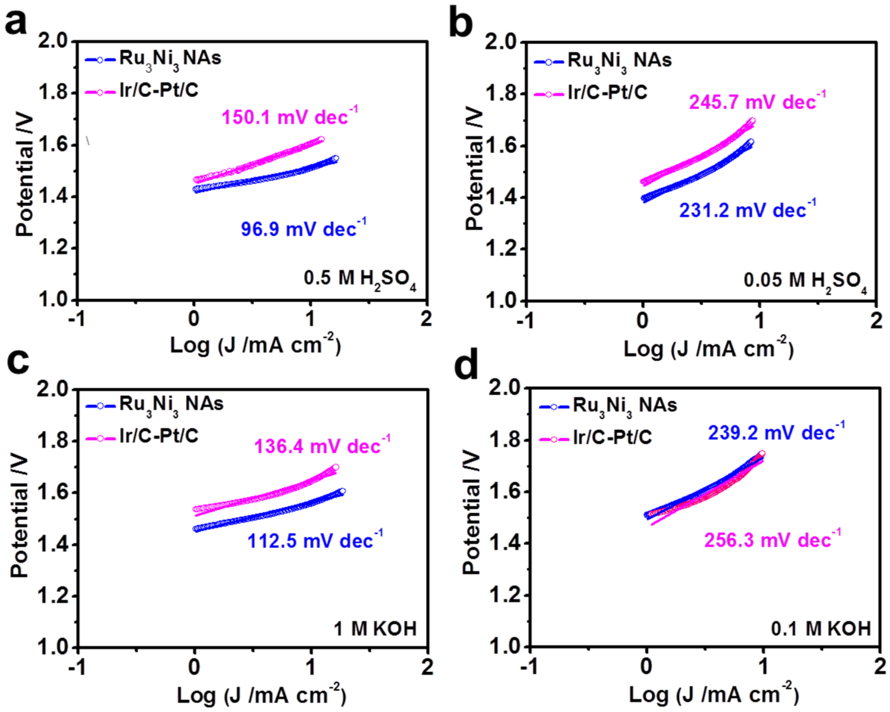

Figure S20. The corresponding water splitting Tafel plots of Ru_3Ni_3 NAs and Ir/C-Pt/C, related to Figure 4.

(a) 0.5 M H_2SO_4, (b) 0.05 M H_2SO_4, (c) 1 M KOH and (d) 0.1 M KOH, derived from Figure 4a.

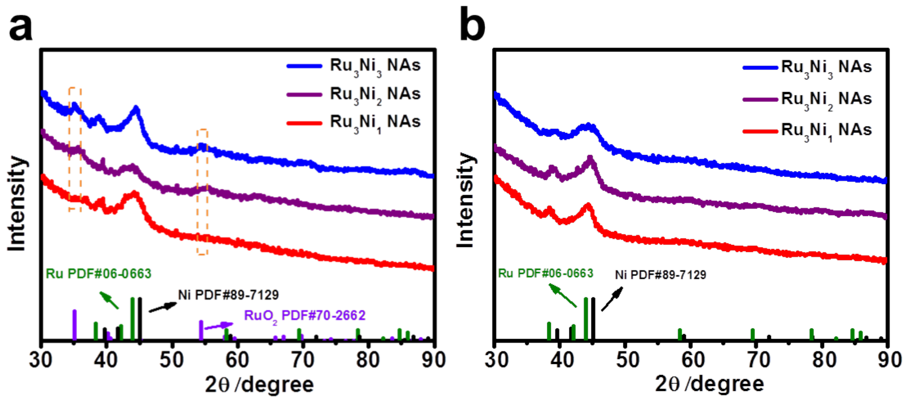

Figure S21. The XRD patterns of Ru_3Ni_3 NAs, Ru_3Ni_2 NAs and Ru_3Ni_1 NAs after heat treatment in air at different temperature and time, related to Figure 5.

(a) 350 °C for 2 h and (b) 250 °C for 1 h.

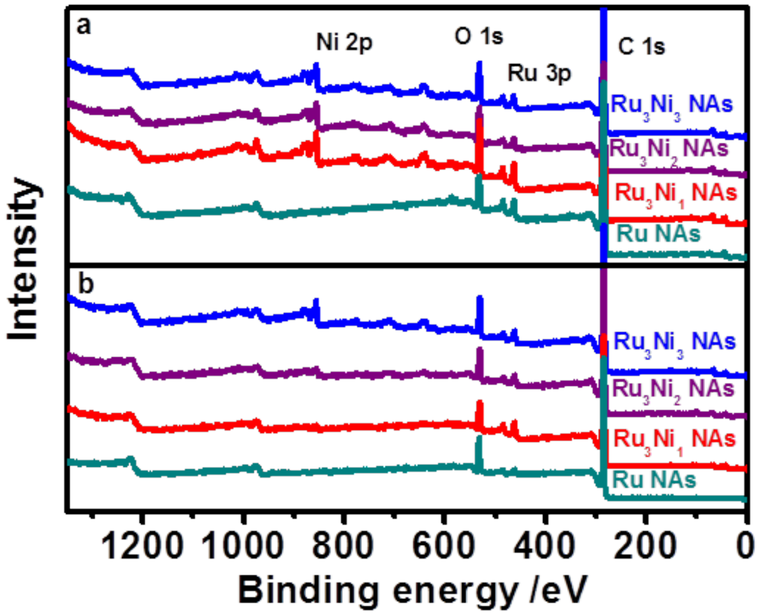

Figure S22. Full scan XPS curves of Ru_3Ni_3 NAs, Ru_3Ni_2 NAs, Ru_3Ni_1 NAs and Ru NAs treated in different environment, related to Figure 5.
(a) at 350 °C in air for 2h and (b) at 250 °C in air for 1h.

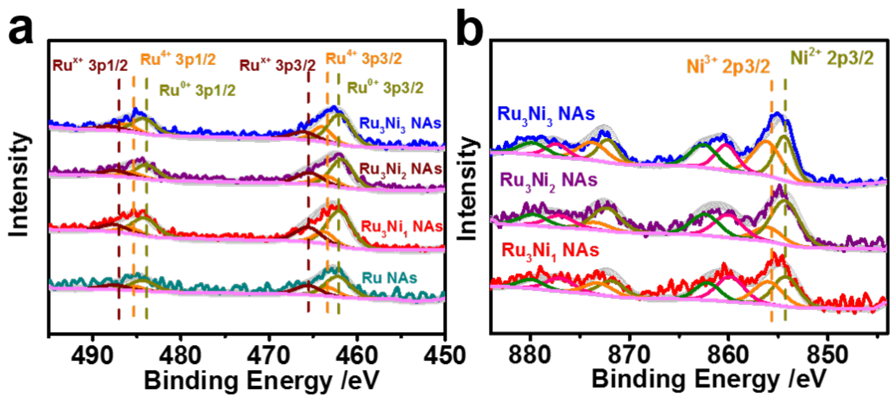

Figure S23. XPS analysis, related to Figure 5.

(a) Ru 3p and (b) Ni 2p curves of Ru_3Ni_3 NAs, Ru_3Ni_2 NAs, Ru_3Ni_1 NAs and Ru NAs treated heated at 250 °C in air for 1h.

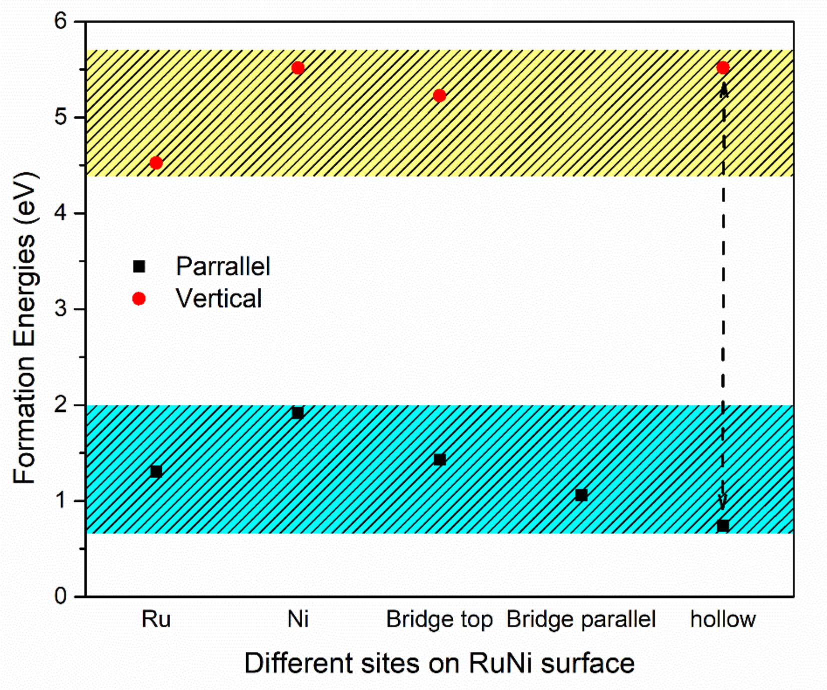

Figure S24. Diagram of absorption energies of absorbed oxygen on RuNi (0001) surface at different sites, related to Figure 6.

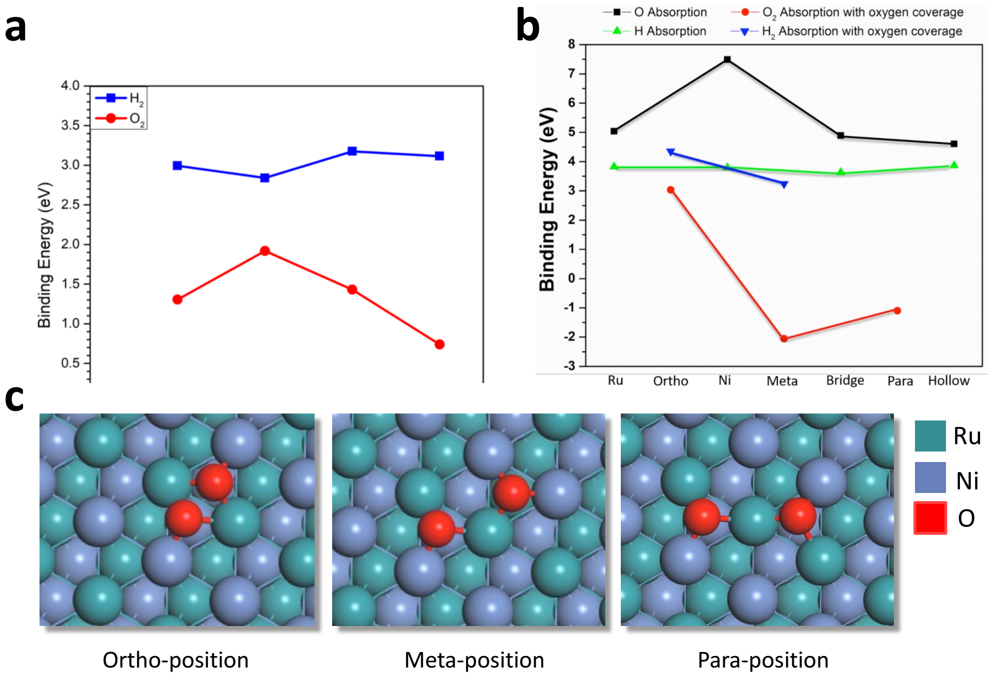

Figure S25. The binding energies diagrams and structural configurations, related to Figure 6.

(a) pristine surface, (b) different partial O-coverage are illustrated, focusing on the cubic phase RuNi (111) surface with considerations on the absorptions of O, H, O_2 and H_2, respectively. (c) Structure configuration of oxygen adsorption.

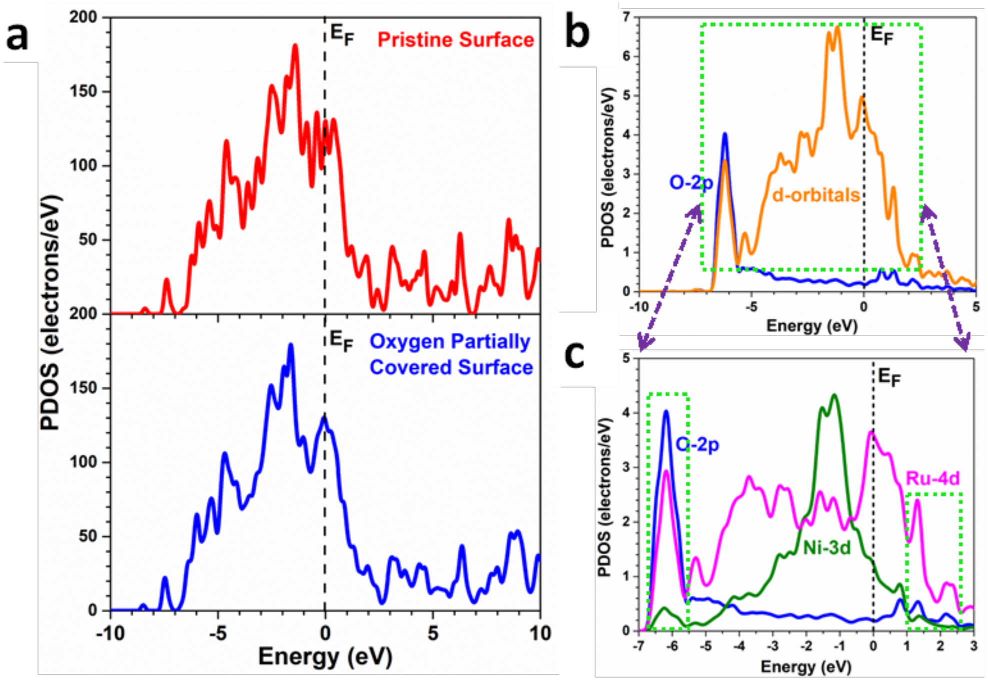

Figure S26. The Comparison of DOS between pristine and oxygen partially covered RuNi surface, related to Figure 6.

(a) Comparison of TDOS between pristine surface and oxygen covered surface. (b) The PDOS of p orbitals from oxygen cover and d orbitals from top surface layer of RuNi model. (c) Illustration of the orbital overlap among O-2p orbitals, Ni-3d orbitals and Ru-4d orbitals.

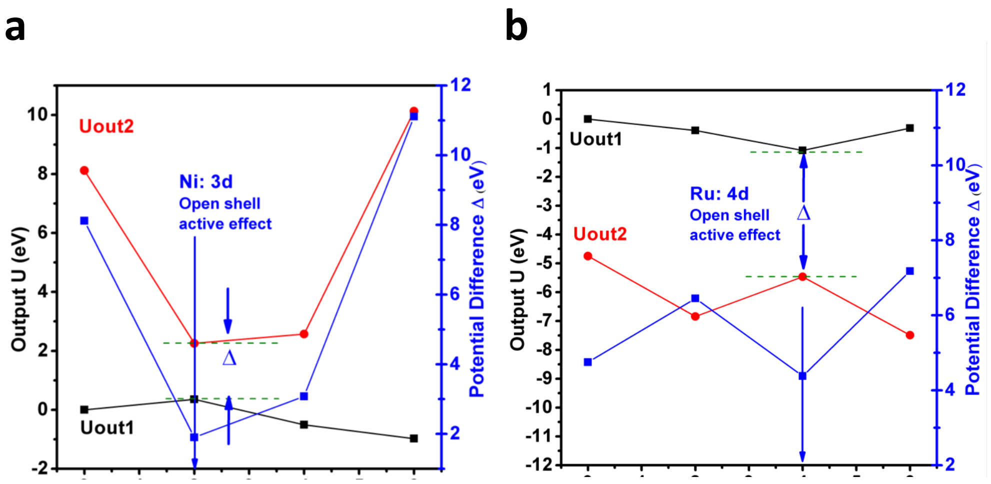

Figure S27. The on-site orbital potential energy projections (Uout1 and Uout2) related to Figure 6.

(a) Ni-3d and (b) Ru-4d on the partially O-covered RuNi surface are demonstrated.

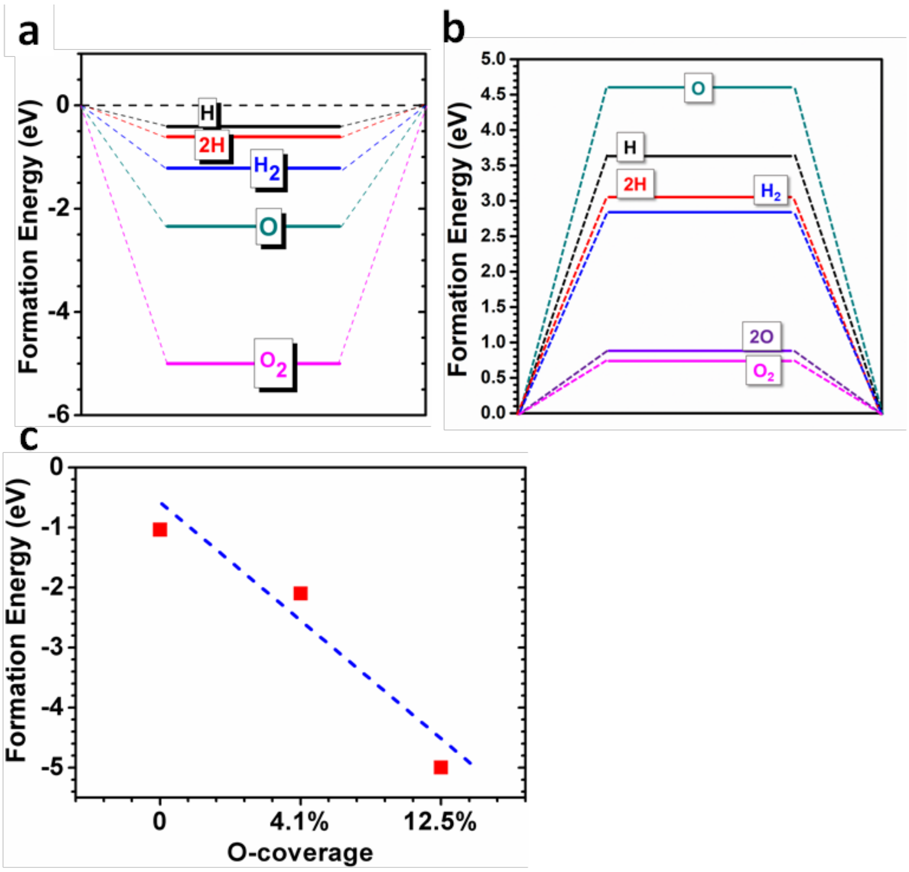

Figure S28. Formation energy of HER and OER, related to Figure 6.

(a) The formation energy for the HER and OER in hexagonal RuNi surface system with partial oxidation states. (b) The chemisorption energy for the HER and OER in cubic phase of RuNi surface system. (c) The O_2 formation energy comparison with different O-coverage on the hexagonal RuNi surface system

Table S1. Summary of activities of HER and OER of Ru-Ni NAs, Ru NAs, Pt/C, Ir/C in alkaline and acidic electrolytes, related to Figure 2 and Figure 3.

			**Ru_3Ni_3 NAs**	**Ru_3Ni_2 NAs**	**Ru_3Ni_1 NAs**	**Ru NAs**	**Pt/C**	**Ir/C**
**1 M KOH**	Overpotential (mV)	**HER**	39	42	44	62	90	—
		**OER**	304	309	301	351	—	311
	Tafel slope (mV dec^{-1})	**HER**	26.9	29.9	30.5	58.3	46.8	—
		**OER**	91.7	67.9	73.4	111.1	—	47.1
**0.1 M KOH**	Overpotential (mV)	**HER**	119	127	123	152	132	—
		**OER**	394	390	384	439	—	407
	Tafel slope (mV dec^{-1})	**HER**	74.4	68.4	57.3	76.0	99.7	—
		**OER**	133.8	131.4	130.2	140.7	—	111.1
**0.5 M H_2SO_4**	Overpotential (mV)	**HER**	39	39	46	55	41	—
		**OER**	252	260	268	277	—	328
	Tafel slope (mV dec^{-1})	**HER**	53.9	53.5	54.2	81.6	29.6	—
		**OER**	45.8	46.1	46.0	71.8	—	64.8
**0.05 M H_2SO_4**	Overpotential (mV)	**HER**	96	115	112	122	109	—
		**OER**	312	313	327	341	—	372
	Tafel slope (mV dec^{-1})	**HER**	67.1	64.0	66.8	79.6	48.8	—
		**OER**	70.8	78.1	82.1	102.3	—	94.1

Table S2. Summary of the recently reported HER electrocatalysts in alkaline and acidic electrolytes. [a]without *i*R compensation. η denotes overpotential, related to Figure 2.

Catalyst	Electrolyte	Current Density	η /mV	Tafel slope (mV/dec)	Ref.
**[a]Ru_3Ni_3 NAs**	**1 M KOH**<br>**0.1 M KOH**<br>**0.5 M H_2SO_4**<br>**0.05 M H_2SO_4**	**10 mA cm^{-2}**	**39**<br>**119**<br>**39**<br>**96**	**26.9**<br>**74.4**<br>**53.9**<br>**67.1**	**This work**
[a]A-Ni-C	0.5 M H_2SO_4	10 mA cm^{-2}	34	41	*Nat. Commun.* **7**, 10667 (2016).
[a]MoSx/NCNT	0.5 M H_2SO_4	10 mA cm^{-2}	110	*40*	*Nano Lett.* **14**, 1228-1233 (2014).
Ru@C_2N	0.5 M H_2SO_4	10 mA cm^{-2}	22	30	*Nat. Nanotechnol.* **12**, 441-446 (2017).
SV-MoS_2	0.5 M H_2SO_4	10 mA cm^{-2}	170	60	*Nat. Mater.* **15**, 48-53 (2016).
FeP NA/Ti	0.5 M H_2SO_4	10 mA cm^{-2}	55	38	*Angew. Chem. Int. Ed.* **53**, 12855-12859 (2014).
$CoMoS_x$	0.1 M $HClO_4$<br>0.1 M KOH	5 mA cm^{-2}	207<br>158	/	*Nat. Mater.* **15**, 197-203 (2016).
$NiFeO_x$/CFP	1 M KOH	10 mA cm^{-2}	88	84.6	*Nat. Commun.* **6**, 7261 (2015).
MoC_x nanooctahedrons	1 M KOH	10 mA cm^{-2}	151	59	*Nat. Commun.* **6**, 6512 (2015).
CoN_x/C	1 M KOH	10 mA cm^{-2}	170	75	*Nat. Commun.* **6**, 7992 (2015).
WC/Ni foam	0.1 M KOH	10 mA cm^{-2}	220	/	*J. Am. Chem. Soc.* **137**, 5480-5485 (2015).
CoFeP	1 M KOH	10 mA cm^{-2}	79	40	*Energy Environ. Sci.* **9**, 2257-2261 (2016).
$Ni(OH)_2$/Pt-islands/Pt(111)	0.1 M KOH	10 mA cm^{-2}	130	/	*Science 334, 1256 (2011)*
β-$Ni(OH)_2$/Pt	1 M KOH	10 mA cm-2	115	42	*ACS Energy Lett. 3, 237 (2018).*

Table S3. Summary of the recently reported OER electrocatalysts in alkaline and acidic electrolytes. [a]without *i*R compensation. η denotes overpotential, related to Figure 3.

Catalyst	Electrolyte	Current Density	η /mV	Tafel slope (mV/dec)	Ref.
**[a]Ru_3Ni_3 NAs**	**0.5 M H_2SO_4**<br>**0.05 M H_2SO_4**<br>**1 M KOH**<br>**0.1 M KOH**	**10 mA cm^{-2}**	**252**<br>**312**<br>**304**<br>**394**	**45.8**<br>**70.8**<br>**91.7**<br>**133.8**	**This work**
$IrO_x/SrIrO_3$	0.5 M H_2SO_4	10 mA cm^{-2}	270	/	*Science* **353**, 1011-1014 (2016).
$IrNiO_x$/Meso-ATO	0.05 M H_2SO_4	10 mA cm^{-2}	320	/	*Angew. Chem. Int. Ed.* **54**, 2975-2979 (2015).
Ir	1 M H_2SO_4<br>1 M KOH	10 mA cm^{-2}	360<br>430	/	*J. Am. Chem. Soc.* **137**, 4347-4357 (2015).
Ru	1 M H_2SO_4<br>1 M KOH	10 mA cm^{-2}	340<br>320	/	*J. Am. Chem. Soc.* **135**, 16977-16987 (2013).
N-doped GM	0.1 M KOH	10 mA cm^{-2}	540	/	*Adv. Mater.* **28**, 6845-6851 (2016).
S, S-CNT	1 M KOH	10 mA cm^{-2}	350	95	*Adv. Energy Mater.* **6**, 1501966 (2016).
N/Co-doped PCPRGO	0.1 M KOH	10 mA cm^{-2}	430	292	*Adv. Funct. Mater.* **25**, 872-882 (2015).

Table S4. The area ratios of Ru^{x+}, Ru^{4+} and Ru^0 in Ru_3Ni_3 NAs, Ru_3Ni_2 NAs, Ru_3Ni_1 NAs and Ru NAs after thermal treatment in air at 350 °C for 2h, derived from Ru 3p XPS results, related to Figure 5.

**Area ratios**	**Ru^{x+} %**	**Ru^{4+} %**	**Ru^0 %**	**Total %**
Ru_3Ni_3 NAs	25.59	57.00	17.41	100
Ru_3Ni_2 NAs	29.32	44.46	26.22	100
Ru_3Ni_1 NAs	26.84	44.57	28.59	100
Ru NAs	24.08	29.89	46.03	100

Table S5. The area ratios of Ru^{x+}, Ru^{4+} and Ru^0 in Ru_3Ni_3 NAs, Ru_3Ni_2 NAs, Ru_3Ni_1 NAs and Ru NAs after thermal treatment in air at 250 °C for 1h, derived from Ru 3p XPS results, related to Figure 5.

**Area ratios**	**Ru^{x+} %**	**Ru^{4+} %**	**Ru^0 %**	**Total %**
Ru_3Ni_3 NAs	16.31	23.95	59.74	100
Ru_3Ni_2 NAs	25.15	18.08	56.77	100
Ru_3Ni_1 NAs	25.58	16.36	58.06	100
Ru NAs	25.21	18.71	56.08	100

Table S6. Absorption energy of different sites on RuNi (0001) surface, related to Figure 6.

Site	Oxygen Position	Formation Energies (eV)	Oxygen Dissociation
Ni Top	parallel	1.918	Yes
	vertical	5.514	No
Ru Top	parallel	1.305	Yes
	vertical	4.526	No
Hollow	parallel	0.739	Yes
	vertical	5.519	No
Bridge top	parallel	1.430	Yes
	vertical	5.227	No
Bridge parallel	parallel	1.057	Yes

-

Table S7. Formation energy of Ru top site on distorted RuNi (0001) surface, , related to Figure 6.

Site	Position	Formation energies (eV)	Oxygen Dissociation
Ru Top	parallel	4.485	No
	vertical	4.811	No

TRANSPARENT METHODS

Materials

Ruthenium (III) acetylacetonate ($Ru(acac)_3$, 97%) was purchased from Sigma-Aldrich. Nickel (II) acetylacetonate ($Ni(acac)_2$, 96%) and polyvinylpyrrolidone (PVP, MW=58000) were provided by J&K Scientific LTD. Benzyl alcohol (C_7H_8O, 99%) was purchased from Sinopharm Chemical Reagent Co. Ltd. (Shanghai, China). Phloroglucinol ($C_6H_6O_3$, 99%) and ethylene glycol (C_2H_7O, 99%) were purchased from Aladdin. All chemicals were used without further purification. The water (18 MΩ cm^{-1}) used in all the experiments was prepared by passing through an ultra-pure purification system (Aqua Solutions).

Preparation of hierarchical Ru-Ni NAs.

In a typical preparation of Ru_3Ni_3 NAs, 10 mg $Ru(acac)_3$, 6.3 mg $Ni(acac)_2$, 75.9 mg phloroglucinol, 5 mg tetramethylammonium bromide, 100 mg PVP and 10 mL benzyl alcohol were added into a 35 mL glass vial. After the vial was capped, the mixture was ultrasonicated for approximately 40 min. The resulting homogeneous mixture was then heated from room temperature to 160 °C and maintained at 160 °C for 5 h in an oil bath. The products were then collected by centrifugation and washed with an ethanol/acetone mixture. The preparations of the Ru_3Ni_2 NAs and Ru_3Ni_1 NAs were achieved by changing the amounts of $Ni(acac)_2$ from 6.3 mg to 4.2 mg and 2.1 mg while keeping the other parameters constant. The Ru NAs was prepared using the same procedure without $Ni(acac)_2$.

Characterizations

The samples were prepared by dropping ethanol dispersions of the samples on carbon-coated copper TEM grids using pipettes and drying them under ambient conditions. Low-magnification transmission electron microscopy (TEM)

was conducted on a HITACHI HT7700 transmission electron microscope applying an acceleration voltage of 120 kV. High-magnification TEM and HAADF-STEM images were conducted on an FEI Tecnai F20 transmission electron microscope with an acceleration voltage of 200 kV. Powder X-ray diffraction (PXRD) patterns were collected by an X'Pert-Pro MPD diffractometer (Netherlands PANalytical) with a Cu Kα X-ray source (λ=1.540598 Å). X-ray photoelectron spectra (XPS) were collected with an SSI S-Probe XPS spectrometer. All spectra used for the calculation of the d-band centre were corrected by a Shirley background. The d-band centres of the different catalysts were obtained by using the equation: $\frac{\int N(\varepsilon)\varepsilon d\varepsilon}{\int N(\varepsilon)d\varepsilon}$ in the range from 0 eV to -9.0 eV. N represents the density of states.

Electrochemical measurements.

A three-electrode system controlled by a CHI 660E electrochemistry workstation was used to carry out the electrochemical measurements. All Ru-Ni NAs with different ratios were loaded on the carbon powder (20% loading). The catalyst (2 mg) was mixed with isopropanol (1 mL) and Nafion (5 μL). A homogeneous ink was obtained after sonication (30 min). The working electrode (diameter: 0.5 cm, area: 0.196 cm^{-2}) was fabricated by casting 10 μL of the catalyst ink onto a glassy-carbon electrode (GCE). The reference electrode was a saturated calomel electrode (SCE). The counter electrode was a carbon rod. No iR compensation was provided through positive feedback using the CHI 660E electrochemistry workstation. Linear sweep voltammetry (LSV) was carried out with a scan rate of 5 mV s^{-1}. The working electrode was held at a constant current density of 5 mA cm^{-2} and 10 mA cm^{-2} for a continuous time, and the operating potential was finally measured as a function of time.

DFT models and calculations.

We used the CASTEP code to perform our DFT+U calculations (Clark et al., 2005). In this framework, we use the rotationally invariant (Anisimov type) DFT+U functional (Vladimir et al., 1997) and the Hubbard U parameter self-consistently determined for the pseudized Ni-3d and Ru-4d orbital by our new linear response method (Huang, 2016), which have been already successfully reflecting the electron-electron Coulomb potential for the semi-core orbitals should be considered when using DFT+U (Huang, 2016; Huang et al. 2016; Huang 2014; Huang, 2016; Huang, 2016). The geometry optimization used the Broyden-Fletcher-Goldfarb-Shannon (BFGS) algorithm through all calculations.

The PBE functional was chosen for PBE+U calculations with cutoff energy of 750 eV, with the valence electron states expressed in a plane-wave basis set. The ensemble DFT (EDFT) method of Marzari et al. (Marzari et al., 1997) is used for convergence. The supercell of buckle hexagonal Ru-Ni surface model was chosen with sizes of 108 atoms (i.e. $Ru_{54}Ni_{54}$) and 6-layer thick. The vacuum thickness is set to be 15 Å. We only allow the top two layers to be varied freely. The reciprocal space integration was performed using the the mesh of 2×2×1(Probert et al., 2013) with Gamma-center-off, which was self-consistently selected for total energy minimization. With these special k-points, the total energy is converged to less than 5.0x10-7 eV per atom. The Hellmann-Feynman forces on the atom were converged to less than 0.001 eV/Å.

As to the pseudopotentials, we use the norm-conserving pseudopotentials which can reflect all-electron behavior for outer shell valence electrons for |S-matrix|=1, unlike the ultrasoft pseudopotentials (Hasnip etal., 2006; Laasonen et al., 1993). Therefore, the non-linear core corrected norm-conserving pseudopotential can provide a better response in DFT+U calculations, especially for the calculations of defects (Huang, 2016). We note that our method actually provides almost identical values of the U parameter

for both norm-conserving and ultrasoft pseudopotentials. This means that the obtained value has an intrinsic physical meaning for the studied materials. Meanwhile, this will help us to reflect all-electron behavior of the valence electrons especially for the subtle effect of the 4d electrons and outter 5s electrons. The Ru and Ni norm-conserving pseudopotentials are generated using the OPIUM code in the Kleinman-Bylander projector form (Kleinman and Bylander, 1982), the non-linear partial core correction (Louie et al., 1982) and a scalar relativistic averaging scheme (Grinberg et al., 2000) are used to treat the spin-orbital coupling effect. For this treatment, we similarly choose non-linear core correction technique for correcting the valence-core charge density overlapping in such heavy fermions elements. In particular, we treated the (4d, 5s, 5p) states as the valence states of both Ru and (3d, 4s, 4p) for Ni atoms. The RRKJ method is chosen for the optimization of the pseudopotentials (Rappe et al.,1982).

For all the electronic states calculations on Ru-Ni model, we use the self-consistent determination for the U correction on the localized d orbitals to correct the on-site Coulomb energy of the electron spurious self-energy (Huang, 2017). By that method, the Hubbard U parameters on the half-filled shell of $4d^7$ orbitals of Ru is self-consistently determined to be U_d=2.01 eV, and U_d=5.39 eV for the Ni-$3d^8$, respectively.

Oxygen dissociation

We have also investigated the surface effect on the oxygen dissociation and systematically summarized the physical absorption energies of oxygen on different sites on RuNi (0001) surfaces in both horizon and vertical positions. The results are shown in **Table S6.** From the table, we can clearly see that based on different position of oxygen, the absorption energy can separate into two ranges (shown in **Figure S24**). For oxygen molecule parallel to the surface, the formation energies vary from 0.739 eV to 1.918 eV while the energy range will increase to 4.526 eV to 5.519 eV for oxygen molecule become vertical to the surface.

Apparently, the RuNi (0001) surface shows much stronger tendency to dissociate oxygen when oxygen molecule is parallel to the surface. Oxygen atoms from dissociated oxygen molecule will locate in the nearest two hollow sites after relaxation, which are usually the most stable site for single atom with strongest binding with nearby three metal atoms. RuNi (0001) surface shows evident distortion for oxygen absorption of vertical positions, representing stronger interaction between the surface and the absorbed oxygen molecule. In particular, Ru shows strong interaction with oxygen atoms due to Ru reflects stronger distortion than Ni. Generally, Ru, Ni and bridge top absorption sites reflect the similar trends of formation energies in both parallel and vertical oxygen positions. However, the hollow site shows a distinct phenomenon on oxygen absorption, which has the lowest formation energy in parallel position but the highest formation energy in vertical position. This is might result from the interaction competition of different nearby metal atoms to the oxygen molecule. Ru exhibits the lowest energy cost in vertical position. Interestingly, we find out that when oxygen molecule is vertical above Ru top site, oxygen molecule will become parallel above Ru under the strong interaction from Ru atom. After relaxation, Ru atom will rise from the surface and form stable

bonds with oxygen molecule. To further confirm the interaction, oxygen models of parallel and vertical positions have been built directly on the RuNi (0001) surface with distorted Ru atom and energy results are shown in **Table S7**.

After relaxation, both positions show stable oxygen molecule on the surface with bonding with the distorted Ru atom. However, the absorption energy of parallel position will significantly increase and become close to the vertical position result. We speculate that Ru will consume the energy to interact with oxygen atoms even in vertical position. After distortion, Ru will be oxidic by the oxygen and lose the ability to further dissociate with oxygen molecules. Therefore, we conclude that Ru will play a dominant role in interacting with oxygen related molecules or intermediates.

Detailed comparison for the preliminary absorption behavior on the cubic-RuNi (111) and hcp-RuNi (001) surface

1. Energetics

Without any oxygen preliminary coverage during the pre-annealing treatment, the hydrogen molecules actually undergo a high barrier of dissociation. However, their binding energy is too high, which means the H_2 on the RuNi confirms to be a good desorption with energy of 2.7 eV in binding with the RuNi surface (**Figure S25)**.

We further simulate the oxygen partially oxidizing behavior on the RuNi during the pre-annealing treatment experimentally. We found the RuNi surface activity obviously high to perform an evident oxygen molecules cleavage, and therefore, further cause the partial oxidation states with high valence state for Ru and Ni existing on the surface (e.g. Ru^{4+} and Ni^{3+}). More importantly, with the view on the energetics, we confirm that the O-coverage could not proceed infinitely large and exhibit an optimal coverage on this surface. This arises because after a partial O-coverage on the RuNi surface, the O-O bond cleavage could not further be carried out. This indicates the oxygen preliminary coverage that form Ru-O and Ni-O bonding could not continuously occur when the O-O bond cleavage is inhibited. Thus, the OER further desorption performance can be guaranteed with pre-annealing treatment in our experiments, which has been confirmed by our DFT calculations.

ccording to our binding energy calculation, we confirm that oxygen cover on the surface can significantly affect the further absorption behaviors. Several possible combinations of oxygen coverage have been built for illustrating the physicochemical trend, which are ortho-, meta-, and para- positions respectively. We found that the energy barrier for O_2 absorption has been largely decrease. However, the O-O bond cleavage has been further prohibited. Even the absorption energy of O_2 molecule may be further reduced,

while the surface activity increase to a saturation level as shown in the Figure X1b. In Meta- and Para- positions, the suppression of O-O bond dissociation in both O_2 and H_2O are successfully achieved with even lower energy barriers. The binding energy of H is approaching nearly constant independent to the absorption-surface or absorption-site preferences, which denoting a saturated level for highly efficient desorption performance in HER.

2. Electronic structures

We further test the difference of the RuNi surface model with and without partial O-coverage effects. As shown in the total density of states (TDOS, **Figure S26)**, the overall system does not show any substantial difference or contrast in the electronic states especially to the surface levels. Only a minor difference has been noted as the absence of the long-range order of the Ru and Ni sites, as the absence of the van-Hove singularities and spectra-smoothing behavior have been simultaneously observed.

We further on a projected partial density of states (PDOSs) on the RuNi surface to interpret the interplay effect, especially to the d-orbitals of Ru and Ni sites, as well as the adsorbing O-2p orbitals. The PDOS shows an obvious overlapping effect between the surface d-band and O-2p orbital level where the O acts as the stable surface adsorbates **(Figure S26**). This denotes the Ru and Ni will be rather active to take an open and active site to locate the extrinsic O absorption via the initial O-O cleavage. This self-consistently confirms our above energetic analysis of the O-partial coverage effect on the RuNi, which is energetically preferable.

With more detail, we projected the d-bands with individual contributions from the Ru and Ni sites on the surface, respectively **(Figure S26)**. It is found that the Ru-4d orbital level has substantially clear overlapping effect with adsorbed O-2p orbitals, while only a minor overlapping effect to the Ni-3d orbital level.

This indicates the physical trend that, there has been an evidently stronger electron transfer between Ru-site and adsorbed O-related species on the RuNi surface, especially to the partially O-covered. This can further explain the Ru exhibits higher valence charge states than the one found in Ni sites, which are Ru^{4+} and Ni^{3+} reported in our experimental section of this manuscript.

3. Orbital energetic behaviors

The electronic properties have been described with recently developed ab-initio orbital corrections (Huang, 2016; Huang, 2016; Huang, 2016). The core of the method reflects a generalized searching path towards the optimal parameters valid to exhibits the different chemical bonding information within various materials systems [2, 3]. Here in the work, total energy calculation can be successfully achieved with consideration of the targeted orbital projected under any given case of bonding.

With the on-site orbital potential energy projections on the partially O-covered RuNi surface, we found the nearest neighboring (next to O) Ru and Ni sites have some evident changes different from the pristine RuNi surface. With existence of the oxidation states, a strong charge transfer between Ru/Ni-sites and O adsorbates has been found. Overall, from the projections **(Figure S27)**, they all show the open-shell active effect which means in the RuNi alloy system, the electrons transfer are very active and easy, which has been previously confirmed by our isotropic metallic band structure based on the hexagonal lattice.

As shown in the projections, the Ni-3d orbital exhibits an open-shell effect where the electronic on-site coulomb potential reduces from 5.39 eV to the 1.72 eV (ΔNi= -3.67 eV) indicating an electronic transfer from the original Ni-site to the others, which is the O-site as we confirmed. Meanwhile, a weakened electronic negativity for the Ni-site has been found **(Figure S27)**.

Further on the behavior for the Ru-4d orbital, it is found that the Ru-4d open-shell effect turns to be wider gap compared to the Ni-3d projection, potentially indicating a stronger electron transfer effect between Ru and O than the Ni sites. The on-site electronic Coulomb potential for the Ru-4d changes from 2.01 eV to the 6.38 eV (ΔRu= +4.37 eV) **(Figure S27)** showing a stronger electronic negativity on the Ru site as it stands for the high capabilities in oxidation by further O-coverage. Therefore, the high valence charge states of the Ru^{4+} and Ni^{3+} can be also reflected from our calculations and the calculation self-consistently confirmed their existence, where correlates to the water splitting performance in our experiment.

4. Adsorption analysis

From **Figure 6c**, it is noted that O shows much lower chemisorption energy than other species on the hexagonal surface that can also prove the oxidation states on the surface will be possible to happen. Moreover, we have operated detailed calculations on the RuNi surface with partial oxidation states. Comparing to H or H_2 or H_2O, the chemisorption of O_2 and O atoms is highly favorable and very stable on the surface, which can support that why the concentration of oxidation states is very high **(Figure S28)**. For better comparison and explanation of why oxidation state occurred, we have also operated detailed calculations on the cubic surface to observe the oxidation states. We found the O adsorption formation energy can be further lowered downshift when more O adsorption participates **(Figure S28)**. Therefore, our calculations on the RuNi surface with and without partial oxidation states have been carried out, which will assist us on the interpretation of the mechanism between microscopic behaviors and macroscopic experimental observations. Particularly in the **Figure S28**, it has shown the formation energies of H, 2H, H_2, O, and O_2 on the RuNi surface with partial oxidation. Even though the total energy is larger than the hexagonal phase, the chemisorption energy tendency

between different species is similar. $2H \rightarrow H_2$ is still energetically favorable that facilitate the HER reaction. Oxygen atoms adsorption near the surface shows different behavior. Note that, the binding energy of single oxygen atom on surface is much higher. However, the chemisorption energy of oxygen atom has been significantly decreased to near 0.7 eV, meaning the strong tendency of further 2O chemisorption. The low absorption energy of O_2 supports both $2O \rightarrow O_2$ and O_2 desorption reactions. Therefore, both hexagonal and cubic phase of such Ru-Ni system shows high catalytic reactivity for water splitting based on DFT calculations.

We further compared the O_2 adsorption energy with related to the different percentage of O-coverage (to quantitatively denote the oxidation state coverage rate), in forms of 0% (without coverage), 4.1% (very light coverage), and 12.5% (light coverage). The adsorption energy of O_2 has shown a clear physicochemical down-trend to stabilize on the surface without any O-O bond cleavage **(Figure S28)**. This indicates the oxidation state will promote the stabilities of O_2 on the surface of RuNi system.

REFERENCES

CLARK, S. J., SEGALL, M. D., PICKARD, C. J., HASNIP, P. J., PROBERT, M. I., REFSON, K & PAYNE, M. C. 2005. First principles methods using CASTEP. *Z Kristallogr*, .220, 567-570

VLADIMIR, I. A., ARYASETIAWAN, F & LICHTENSTEIN, A. I. 1997. First-principles calculations of the electronic structure and spectra of strongly correlated systems. *J Phys Condens Matter* 9, 767.

HUANG, B., GILLEN, R & ROBERTSON, J. 2014. Study of CeO_2 and its native defects by density functional theory with repulsive potential. *J Phys Chem C*, 118, 24248-24256.

HUANG, B. 2014. Superiority of DFT+ U with non-linear core correction for open-shell binary rare-earth metal oxides: a case study of native point defects in cerium oxides. *Philos Mag*, 94, 3052-3071.

HUANG, B. 2016. Unraveling energy conversion modeling in the intrinsic persistent upconverted luminescence of solids: a study of native point defects in antiferromagnetic Er_2O_3. *Phys Chem Chem Phys*, 18, 13564-13582.

MARZARI, N., VANDERBILT, D & PAYNE, M. C. 1997. Ensemble density-functional theory for ab initio molecular dynamics of metals and finite-temperature insulators. *Phys Rev Lett*, 79, 1337.

PROBERT M. I. J & PAYNE, M. C. 2003. Improving the convergence of defect calculations in supercells: An ab initio study of the neutral silicon vacancy. *Phys Rev B*, 67, 075204.

HASNIP P. J., PICKARD, C. & COMPUT, J. 2006. Electronic energy minimisation with ultrasoft pseudopotentials. *Phys Commun*, 174, 24-29.

LAASONEN, K., PASQUARELLO, A., CAR, R., LEE, C & VANDERBILT, D. 1993. Car-Parrinello molecular dynamics with Vanderbilt ultrasoft pseudopotentials. *Phys Rev B*, 47, 10142.

KLEINMAN L & BYLANDER, D. M. 1982. Efficacious form for model pseudopotentials. *Phys Rev Lett*, 48, 1425.

LOUIE, S. G., FROYEN, S & COHEN, M. L. 1982. Nonlinear ionic pseudopotentials in spin-density-functional calculations. Phys. Rev. B. 26, 1738.

GRINBERG, I., RAMER, N. J & RAPPE, A. M. 2000. Transferable relativistic dirac-slater pseudopotentials. *Phys Rev B*, 62, 2311.

RAPPE, A. M., RABE, K. M., KAXIRAS, E & JOANNOPOULOS, J. D. 1990. Optimized pseudopotentials. *Phys Rev B*, 41, 1227.

HUANG, B. 2017. The screened pseudo-charge repulsive potential in perturbed orbitals for band calculations by DFT+ U. *Phys Chem Chem Phys*, 19, 8008-8025.

HUANG, B. 2016. Intrinsic deep hole trap levels in Cu2O with self-consistent repulsive Coulomb energy. *Solid State Commun*, 230, 49-53.

HUANG, B. 2016. Strong compensation hinders the p-type doping of ZnO: a glance over surface defect levels. *Solid State Commun*, 237, 34-37.

HUANG, B. 2016. 4f fine-structure levels as the dominant error in the electronic structures of binary lanthanide oxides. J Comput Chem, 37, 825-838.
